# Recruitment kinetics of the homologous recombination pathway in procyclic forms of *Trypanosoma brucei* after ionizing radiation treatment

**DOI:** 10.1038/s41598-018-23731-6

**Published:** 2018-03-29

**Authors:** Paula Andrea Marin, Marcelo Santos da Silva, Raphael Souza Pavani, Carlos Renato Machado, Maria Carolina Elias

**Affiliations:** 1 Cell Cycle Laboratory (LECC) - Center of Toxins, Immune Response and Cell Signaling (CeTICS), Butantan Institute, São Paulo, São Paulo, 05503-900 Brazil; 2 Biochemical and Immunology Department, Institute of Biomedical Science, ICB, Federal University of Minas Gerais (UFMG), Minas Gerais, Belo Horizonte, 31270-901 Brazil

## Abstract

One of the most important mechanisms for repairing double-strand breaks (DSBs) in model eukaryotes is homologous recombination (HR). Although the genes involved in HR have been found in *Trypanosoma brucei* and studies have identified some of the proteins that participate in this HR pathway, the recruitment kinetics of the HR machinery onto DNA during DSB repair have not been clearly elucidated in this organism. Using immunofluorescence, protein DNA-bound assays, and DNA content analysis, we established the recruitment kinetics of the HR pathway in response to the DSBs generated by ionizing radiation (IR) in procyclic forms of *T. brucei*. These kinetics involved the phosphorylation of histone H2A and the sequential recruitment of the essential HR players Exo1, RPA, and Rad51. The process of DSB repair took approximately 5.5 hours. We found that DSBs led to a decline in the G2/M phase after IR treatment, concomitant with cell cycle arrest in the G1/S phase. This finding suggests that HR repairs DSBs faster than the other possible DSB repair processes that act during the G1/S transition. Taken together, these data suggest that the interplay between DNA damage detection and HR machinery recruitment is finely coordinated, allowing these parasites to repair DNA rapidly after DSBs during the late S/G2 proficient phases.

## Introduction

*Trypanosoma brucei* is a single-celled digenetic extracellular parasite and the etiological agent of human African trypanosomiasis (sleeping sickness), which almost invariably progresses to death unless treated. This disease is present in many countries in sub-Saharan Africa, which is the poorest region worldwide, causing approximately 70,000 deaths per year^1,2^. This organism replicates in the blood and tissue fluids of mammals (as bloodstream forms) and in the digestive system of tsetse flies (often as procyclic forms). During their migration towards the salivary glands, the procyclic forms differentiate into mesocyclic or epimastigote forms, and in the salivary glands, epimastigotes transform into non-replicative metacyclic forms that can infect mammals^3^. During their transition in different environments and due to their frequent replication, DNA lesions often form that can lead to genomic instability. Among the different types of lesions that occur in DNA, double-strand DNA breaks (DSBs) are the most important because they can have hazardous effects on the cell and are a source of recombination, which is essential during the life cycle of this parasite^4,5^. Although studies have shown that DSBs trigger a robust DNA damage response in *T. brucei*^4,6^, the duration of this process as well as the recruitment kinetics of the primary proteins involved in this process are not well established.

Model eukaryotes have two major repair pathways to ameliorate DSBs and ensure genomic integrity: homologous recombination (HR) and non-homologous end joining (NHEJ). The HR machinery is restricted to the late-S and G2 phases, while the NHEJ machinery is available during all phases of the cell cycle and is predominant during the G1 phase^7–9^. An alternative NHEJ pathway termed microhomology-mediated end joining (MMEJ), or alt-NHEJ, has been reported to play an important role together with HR in DSB repair, primarily during the G1 and S phases, including in trypanosomatids^10–13^. However, some of the important genes involved in the canonical NHEJ pathway (DNA ligase IV and XRCC2) were not found in the *T. brucei* genome database (TriTrypDB), strongly suggesting that this repair mechanism is absent or that it mechanistically diverged in this organism^14,15^. However, genes involved in HR were found, although some of these genes code for proteins that exhibit low shared identities with those in mammals, such as RPA-1, which lacks the N-terminal RPA70N domain that is involved in protein-protein interactions, and it is important for the activation of the ATR signaling pathway in mammalian cells^16,17^.

In general, the HR process in model eukaryotes involves the activation of checkpoint pathways dependent on ataxia-telangiectasia-mutated (ATM) and ataxia telangiectasia and Rad3-related protein (ATR) kinases that promote cell cycle arrest, providing sufficient time for DNA repair^18–20^. After this damage, DSBs are recognized by the complex formed by the proteins MRE11, Rad50, and Nbs1/Xrs2 (MRN or MRX in yeast), which initiates DNA resection^21^. This initial response recruits ATM, inducing its auto-phosphorylation, which activates the checkpoint pathway^22^. The phosphorylation of the variant histone H2AX (in humans) by ATM is then necessary for the assembly of the DNA damage response complex (mediator-MRN-ATM) at damaged sites^23–25^. Additional terminal end DNA processing by specialized nucleases such as EXO1 is necessary for the generation of a longer single-stranded DNA (ssDNA) overhang, which is then coated with the ssDNA-binding complex replication protein A (RPA). RPA bound to DNA recruits an ATRIP mediator to activate the ATR kinase. The complete ATR activation, which is mediated by DNA topoisomerase 2-binding protein 1 (TOPBP1), allows the transfer of DNA filaments from RPA to RAD51 protein. RAD51 transference requires other factors such as RAD51 paralogs, RAD52, and other auxiliary proteins. Thus, the presynaptic complex composed of RAD51 and breast cancer 2 protein (BRCA2) filaments begins strand invasion, searching for homologous sequences and promoting DNA recombination^7,26^.

Several findings point to a canonical HR pathway in *T. brucei*, at least in the bloodstream forms^4,27^. A study using induced high-efficiency meganuclease-mediated DSBs indicated that DSBs led to an accumulation of phosphorylated histone H2A (γH2A) and RAD51 foci as well as a delay in the S and G2 phases of the cell cycle^28^. In addition, several studies indicate the participation of RAD51 during HR in *T. brucei* as well as its interactions with the protein regulator BRCA2^29–31^. While prior studies identified some of the proteins that participate in the HR pathway, the detailed reaction and recruitment kinetics of the primary HR players onto DNA during DSB repair are not clearly understood.

This paper addresses the recruitment kinetics of the HR pathway in response to the DSBs generated by ionizing radiation (IR) in procyclic forms of *T. brucei*. Exo1 bound to DNA appeared during the first half hour after IR treatment. RPA1 ligation onto DNA reached its maximum at 1.5 h after IR, followed by a decrease in Exo1 bound to DNA until two hours after IR, increasing the γH2A intensity that occurred together with RPA1 recruitment, with maximum phosphorylation only observed after the maximum RPA-DNA interaction. When the RAD51-DNA interaction reached its maximum (2–5 h after IR), a gradual γH2A dephosphorylation was observed. The complete process of DSB repair took approximately 5.5 hours, as demonstrated by the return of DNA-bound RAD51 and γH2A to basal levels. We also found that IR-induced DSBs led to a declining G2/M phase 5–6 h after DNA damage, while G1/S arrest was maintained. This finding suggests that HR, which occurs predominantly during the late S/G2 phase, repairs the DSBs more rapidly than other DNA damage response pathways, such as MMEJ, that are supposed to be acting during the G1/S transition. Taken together, these data suggest that the interplay between DNA damage detection and HR machinery recruitment is finely coordinated in *T. brucei* procyclic forms, with *T. brucei* showing HR players that exhibit low identity with those in model eukaryotes.

## Results

### Impact of ionizing radiation on *T. brucei* survival

**Figure 1:**
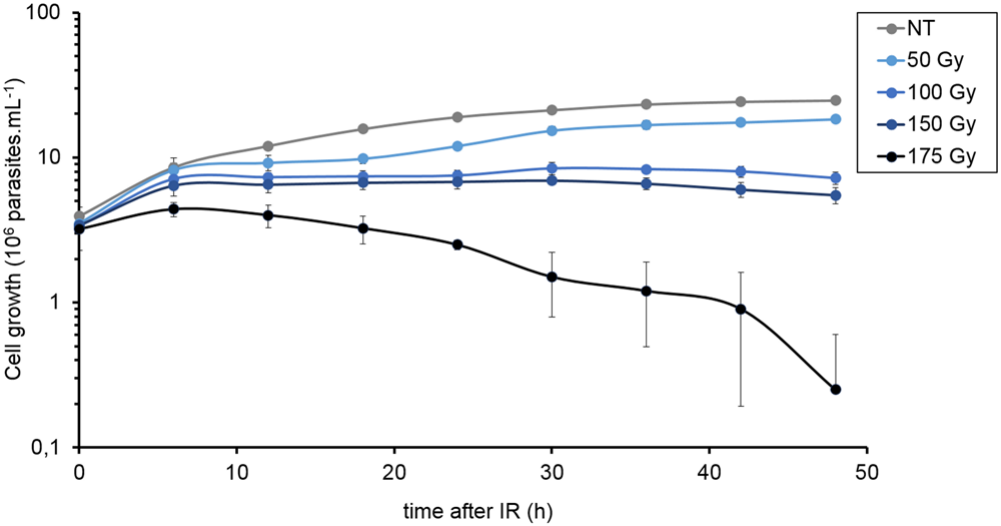
Cell reversibility afterIR treatment. The graph shows the cell growth every 12 h up to 48 h after being exposed to different doses of IR. The data represent the average of three independent experiments, and the error bars represent the standard deviations.

To study the recruitment kinetics of the HR repair pathway in a non-synchronized *T. brucei* procyclic cell culture, we first adjusted the DNA damage treatment. We subjected the parasites to different doses of IR (50, 100, 150, and 175 Gy) to establish the minimum dose that could arrest cell growth reversibly (Fig. 1). We observed that 50, 100, and 150 Gy doses stopped cell proliferation six hours after treatment. Moreover, 50 Gy of IR led to a significant arrest of cell proliferation that was recovered 24 h after IR exposure, suggesting that 50 Gy-induced DNA damage could be repaired.

### IR causes DNA fragmentation and the phosphorylation of histone H2A

**Figure 2:**
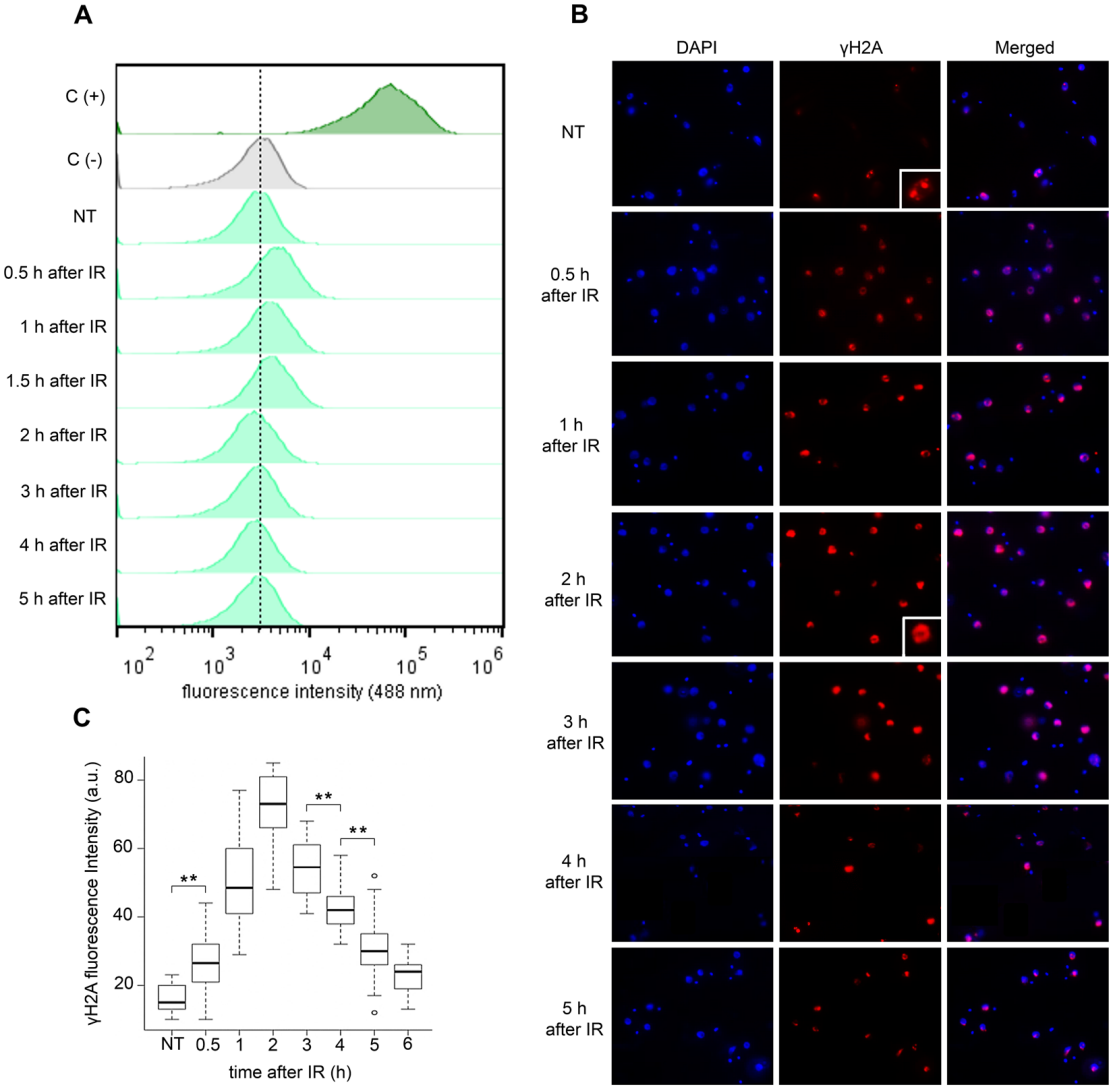
DNA fragmentation detection and H2A phosphorylation after IR. The parasites were treated with 50 Gy of IR and (**A**) the DNA fragmentation was measured using terminal deoxynucleotidyl transferase (TdT-TUNEL) after treatment. C(−) represents a negative control in which the TdT enzyme was not present. In C(+) the samples were pre-treated with DNase I generating therefore the maximum positive fluorescence intensity signal. Dotted line was used to indicate the peak that means negative fluorescence. Therefore, peak to the right of dotted line was considered positive. (**B**) The cells were analyzed by IIF to check the γH2A fluorescence intensity after IR treatment. (**C**) Box plots represent the measurement of the γH2A fluorescence intensity in the IIF assay. The data represent the average for 100 analyzed cells. Bars represent the standard deviation. (**) Indicate significant differences compared to the non-treated cells via a Friedman and Wilcoxon test.

To determine whether IR treatment led to DNA strand breaks, a terminal deoxynucleotidyl transferase dUTP nick end-labeling (TUNEL) assay was performed according to protocols that were already standardized in trypanosomatids^16,32,33^ (see the material and methods section). After IR treatment, the parasites were TUNEL labeled, and their fluorescence intensity was quantified by flow cytometry. In the sample from negative control the enzyme TdT was not added and therefore no signal was expected. It means that the peak of negative control should not be considered as positive signal. In the sample called positive control DNA break was generated treating cells with DNAse to guarantee generation of high amount of DNA break. Therefore, maximum fluorescence intensity signal was observed. Any signal between peaks of negative and maximum ones should be considered as positive TUNEL that means the occurrence of DNA break. Positive TUNEL signals were observed during the first 1.5 hours after IR treatment (Fig. 2A), suggesting that IR exposure generates DSBs in *T. brucei* just as it does in other eukaryotes^34^.

DSBs have already been demonstrated to trigger the phosphorylation of H2A in *T. brucei*^28^. Therefore, to confirm the presence of DSBs after IR treatment, we investigated the presence of γH2A over time after treatment using an anti-γH2A antibody in an indirect-immunofluorescence (IIF) assay (Fig. 2B). A significant positive signal was obtained in treated cells compared to that in non-treated parasites during the first six hours after IR exposure (Wilcoxon test, p = 3.94e^−12^) (Fig. 2B,C). A higher signal was observed two hours after treatment (Fig. 2C). Note that we observed γH2A signals in non-treated (control) parasites, but while the signals detected in the controls were distributed over small foci, the γH2A fluorescence in IR-treated parasites was dispersed throughout the nucleus (Fig. 2B, see zoom images). The dispersed pattern observed after IR is probably due to the generalized DSBs caused by IR, which is different from the punctuated pattern already shown in bloodstream forms after treatment with MMS or phleomycin or in those digested with I-SceI^35^.

### Replication protein A (RPA1) expression increases after IR

Because our results suggested that DNA damage reaches its maximum two hours after IR treatment, we tested whether damaged DNA was repaired during and after this period. To detect DNA repair, we first decided to investigate the behavior of RPA1, the major subunit of the RPA complex. RPA is the major single-stranded binding protein in eukaryotes, and it is a fundamental component of many DNA repair pathways^36^. In fact, we have already demonstrated that RPA is important for DNA repair in *Trypanosoma cruzi*, assuming a nuclear punctuated pattern after UV and hydroxyurea treatment compared with a dispersed nuclear pattern in control cells^37^.

**Figure 3:**
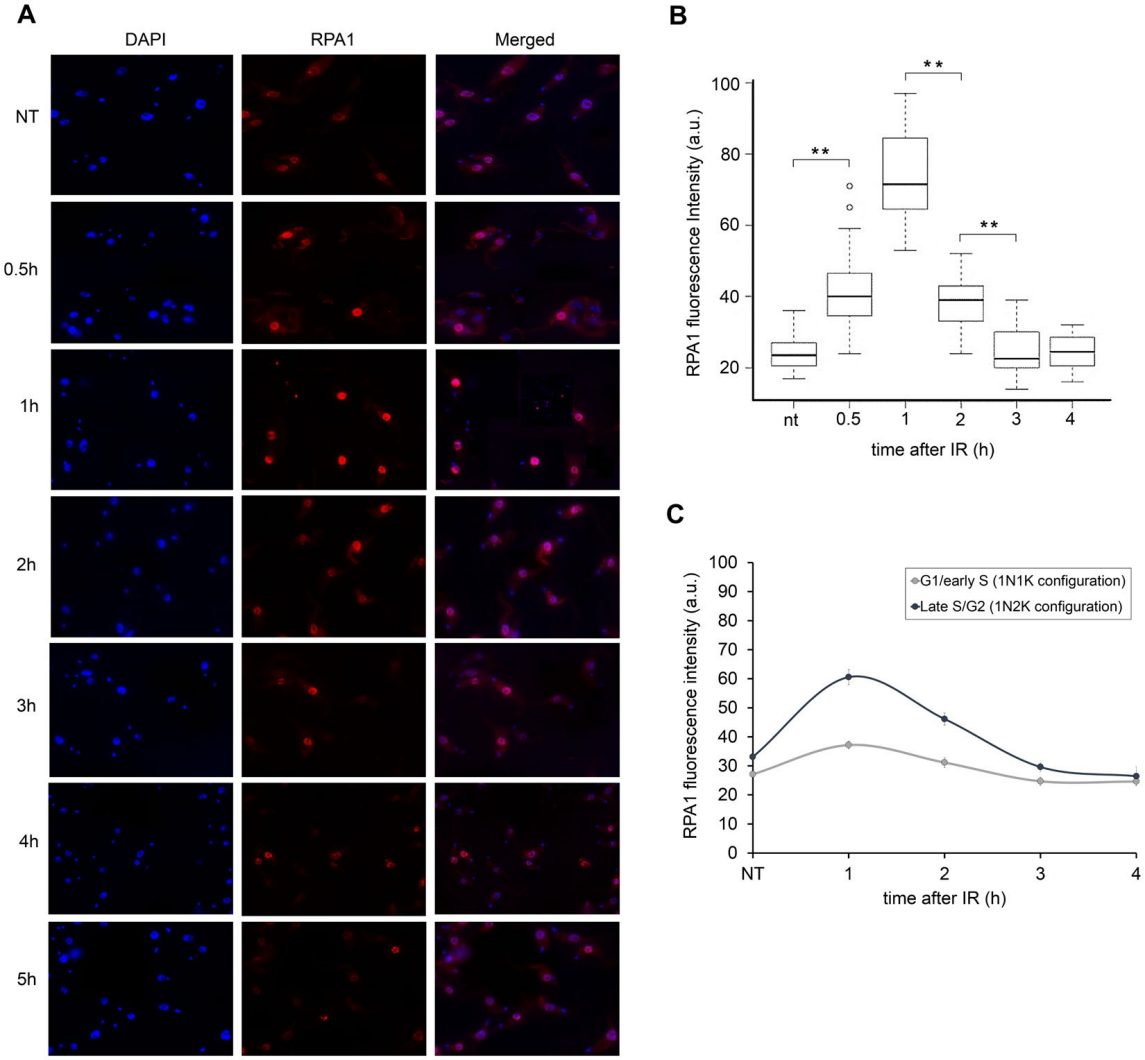
Replication protein A (RPA1) expression increases after IR. The parasites were treated with 50 Gy of IR. (**A**) Immunofluorescence using anti-RPA1 at different time points after treatment. (**B**) Box plots representing the RPA1 fluorescence intensity of the IIF assay presented in A. The data represent the averages of 100 analyzed cells, and the bars represent the standard deviation. (**) Indicate significant differences compared to the non-treated cells as determined by the Friedman and Wilcoxon tests. (**C**) Fluorescence intensity of RPA1, according to the cell cycle phase (G1/early S and late S/G2). The cell cycle phases were estimated based on the duplication of the kinetoplast (K) and nucleus (N) during the cell cycle^62,63^. Data represent the average of 50 analyzed cells for the G1/S pattern and 15 analyzed cells for the late S/G2 pattern.

Thus, we used antisera against RPA1^37^ and performed a fluorescence intensity analysis after DNA damage. When compared to the non-treated parasites, the fluorescence intensity of RPA1 increased in the nucleus from 30 min to two hours after IR treatment (Wilcoxon test, p = 2.41e^−13^) with a maximum peak observed one hour after IR treatment (Fig. 3A,B). We also measured the fluorescence intensity of RPA1 according to the cell cycle phases (G1/early S and late S/G2). We observed that the predominant peak only occurs in late S/G2 (Fig. 3C). Thus, we can speculate that the pathway responsible for repairing DSB in G1/early S, if it exists, is less dependent (or independent) on RPA1.

### HR pathway proteins are upregulated after IR treatment

**Figure 4:**
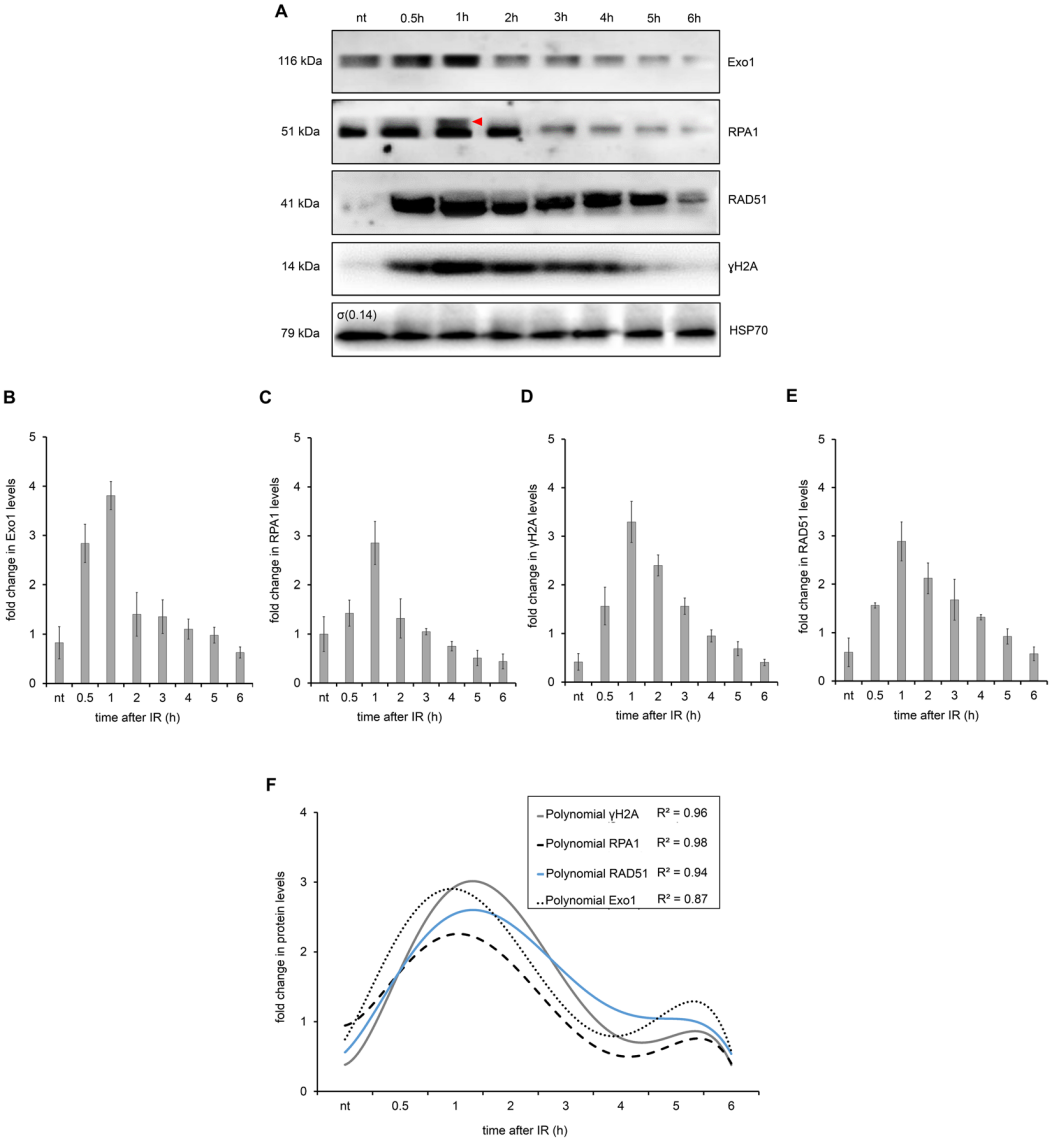
Homologous Recombination (HR) repair proteins in total protein extract after IR. (**A**) WB analysis of the total protein extract after 50 Gy of IR exposure. HSP70 protein was used as an internal control. The red triangle on the RPA1 WB represents a possible RPA1 protein modification. (**B**–**E**) A quantification of the relative intensity of Exo1 (**B**), RPA1 (**C**), γH2A (**D**) and RAD51 (**E**) protein after IR treatment. The data represent the averages of three independent experiments, and error bars represent the standard deviation. (**F**) The sixth degree polynomial regression of WB quantifications. Non-parametric estimation curves and the R2 factor are presented. Notably, the Western blot membranes were cropped to avoid cross-reactions between secondary antibodies during the revealing process.

To continue analyzing the level of protein expression in response to the DSBs caused by IR, we then investigated the levels of some proteins involved in the canonical HR pathway. Therefore, in addition to γH2A and TbRPA1, we monitored the levels of the TbExo1 and TbRAD51 proteins over six hours after IR treatment. The presence of these proteins indicates DSB repair by HR^29^. Lysates from parasites exposed to IR were compared to lysates from non-treated parasites by Western blot (WB) assays (Fig. 4A). The quantification of the WB band intensities for TbExo1, TbRPA1 and TbRAD51 showed that they reached maximum expression levels one hour after IR treatment, corresponding to the increased TbRPA1 signal intensity observed in Fig. 3 (Fig. 4B,E).

Importantly, similar to the γH2A fluorescence intensity in the IIF assay, the total lysate from non-treated parasites exhibited basal levels of γH2A in the WB assay, suggesting a natural genomic instability. Similarly, the WB assay also exhibited basal levels of TbRAD51 and TbExo1 in the total lysate from non-treated parasites. Curiously, a double band of TbRPA1 was observed one hour after IR treatment (Fig. 4A), which we speculate to be the result of a post-translational modification. In summary, the WB measurements indicate that all the repair proteins analyzed here were upregulated approximately one hour after IR treatment. In attempting to better adjust the window of observation for the sequential recruitment kinetics of these proteins, we performed a non-parametric approximation of WB quantification curves. Sixth-degree polynomial curves differentiated among the relative protein levels between 45 min and 1.5 h after IR treatment (Fig. 4F).

### Homologous recombination machinery is rapidly recruited onto DNA after IR

**Figure 5:**
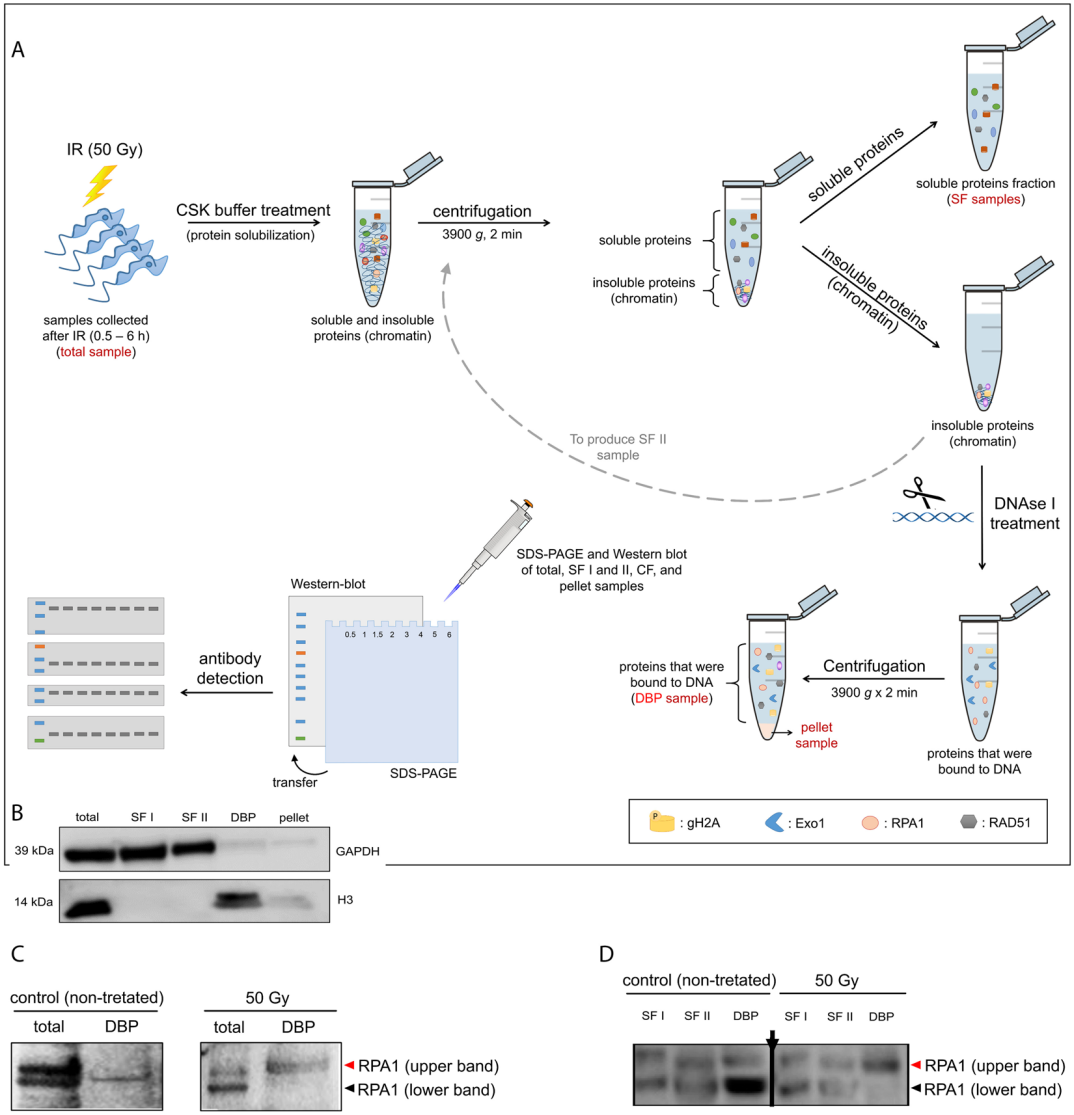
Fractionation assay and WB analysis of RPA1 bands after IR. (**A**) Scheme showing the primary steps of the fractionation assay. The names indicated in red represent the fractions used in WB analysis. (**B**) The GAPDH protein was used as an internal control for the soluble protein fraction (SFI and II). The H3 protein was used as an internal control for the DNA-bound protein fraction (DBP). Western blot membranes were cropped to avoid cross-reactions between secondary antibodies during the revealing process. (**C** and **D**) Molecular weight migration profile of RPA1 in total, soluble protein fraction (SFI and II) and DNA bound protein (DBP) extracts after IR treatment. Arrow in panel D indicates a region where a lane was excluded from the gel, but all samples were analyzed in an unique gel.

**Figure 6:**
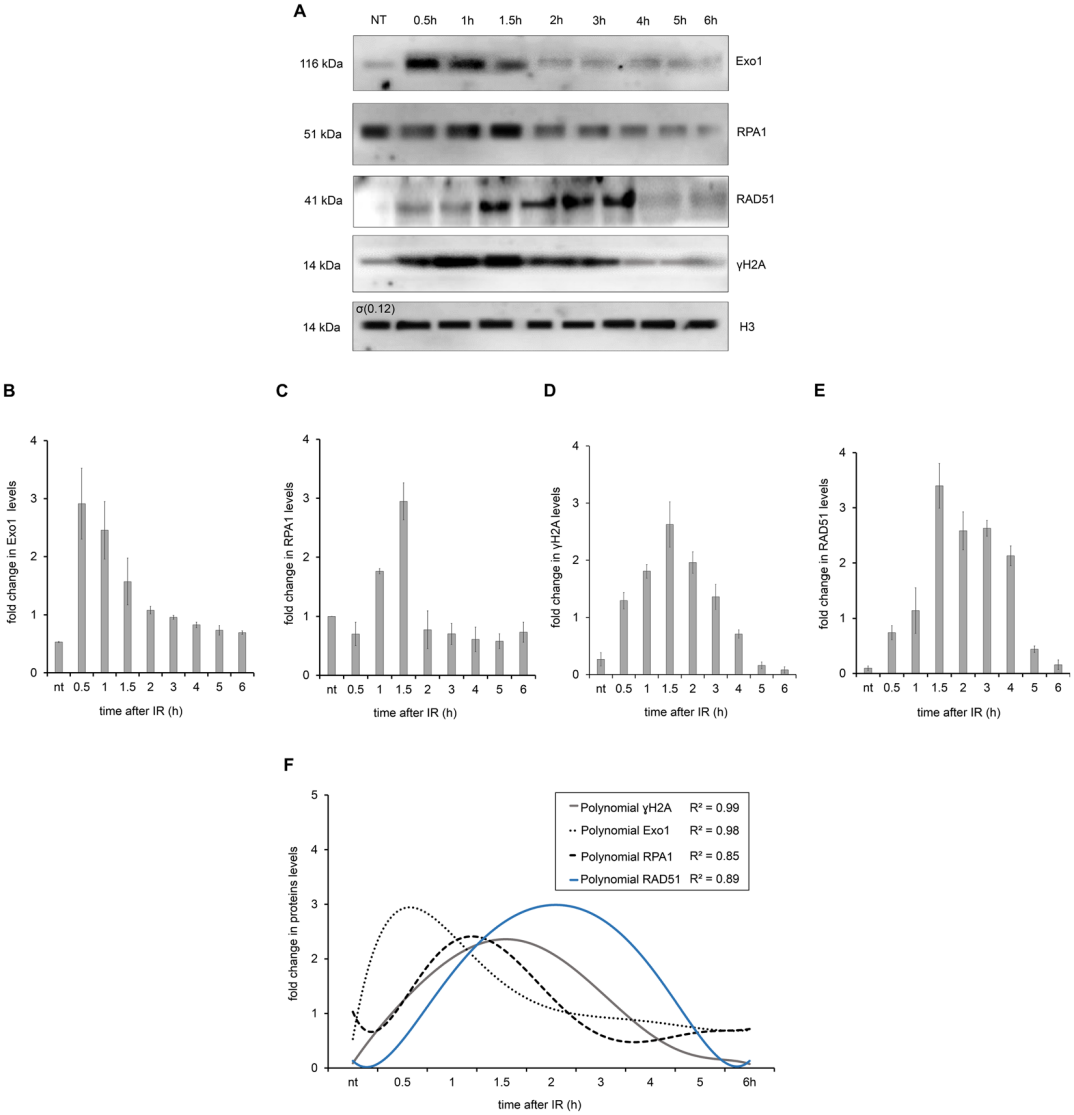
Recruitment of Homologous Recombination (HR) repair proteins to DNA after IR. (**A**) WB analysis of DNA-bound proteins (DBP) derived from the fractionation assay after IR treatment. The H3 protein was used as an internal control. (**B**) A WB quantification of the Exo1 relative intensity after IR treatment. (**C**) A WB quantification of the RPA1 relative intensity after IR treatment. (**D**) A WB quantification of the γH2A relative intensity after IR treatment. (**E**) WB quantification of the RAD51 relative intensity after IR treatment. The data represent the average of three independent experiments and the error bars represent the standard deviations. (**F**) A sixth-degree polynomial regression of the WB quantifications. Non-parametric estimation curves and R2 factors are presented. Notably, the Western blot membranes were cropped to avoid cross-reactions between secondary antibodies during the revealing process.

The upregulation in protein expression after IR does not mean that these proteins have been working on DNA repair. Therefore, we decided to evaluate whether TbExo1, TbRPA1 and TbRAD51 were recruited to the DNA after IR treatment. To distinguish between the soluble and DNA-bound proteins, we prepared fractionated protein extracts to isolate DNA-bound proteins from non-treated and IR-treated parasites (Fig. 5A). This protocol involves the extraction of soluble proteins after permeabilization and then the extraction of DNA-bound proteins after digesting DNA with DNase I (Fig. 5A,B). Because two RPA1 bands were detected in the total extract from *T. brucei* (Fig. 4A), but only one RPA1 band was found in the DNA-bound fraction (Fig. 6A), we studied which of the two RPA1 bands interacts with the DNA. To answer this question, the total extract and the DNA-bound protein fraction from samples obtained one hour after IR were run on the same SDS-PAGE gel. In non-treated cells, the lower molecular weight RPA1 band was bound to DNA, while the higher molecular weight RPA1 band was bound to DNA after IR treatment (Fig. 5C). To better visualize upper and lower RPA1 isoforms; to see whether these isoforms are predominant or exclusive in each fraction and to show the presence of isoforms that are not bound onto DNA in soluble protein fraction we run another SDS-PAGE applying a high amount of sample and allowing a better resolution in the region of RPA1 isoforms. In total extracts both RPA isoforms are present in non-treated and in IR exposed cells. Both isoforms were also found in soluble protein fraction of non-treated and treated cells. However, lower molecular weight RPA1 was the predominant band bound to DNA in non-treated cells while the higher molecular weight RPA1 is the predominant band bound to DNA after IR treatment (Fig. 5D). As an internal control of this assay, we showed that the GAPDH protein was obtained in the soluble fractions (SFI and II), while histone H3 was obtained in the DNA-bound protein fraction (DBP) (Supplementary Fig. 1). The WB quantification of DNA-bound proteins revealed increased levels of DNA-bound TbExo1 30 min after IR, followed by a gradual increase in DNA-bound TbRPA1 and the phosphorylation of H2A (Fig. 6A–D). The maximum intensity of DNA-bound TbRPA1 was achieved 1.5 h after IR, at approximately the same time as the decrease in TbExo1 levels (Fig. 6A,B,C). Two hours after IR treatment, the intensity of DNA-bound TbRPA1 decreased, and the level of γH2A reached its maximum. Notably, we do not know which kinase phosphorylates H2A initially, although based on these kinetics, the γH2A intensity is likely dependent on ATR as a result of RPA recruitment. A control showing the fractionation of soluble and DNA bound proteins of assay presented in Fig. 6A was obtained to check the presence of GAPDH and histone H3 in all the fractions, and it is presented in Supplementary Fig. 2.

Concomitant with a reduction in the presence of DNA-bound TbRPA1 two hours after IR, the DNA-bound TbRAD51 increases (Fig. 6A,E). Together with DNA-bound TbRAD51 (i.e., 2–5 h after IR), we observed the gradual dephosphorylation of γH2A. Thus, we hypothesize that the complete process of DSB repair by HR took approximately 5.5 hours, as demonstrated by the release of TbRAD51 from the DNA, together with the decline in the γH2A signal on the DNA (Fig. 6F). However, the growth impairment from 6 to 18 h after IR shown in Fig. 1 raises a question about why *T. brucei* cells stop growing if the DNA damage has already been repaired. We then hypothesized that there is another checkpoint that acts in response to the presence of DSBs in another cell cycle phase, which lasts longer than HR and is less dependent on (or independent of) the proteins used here, i.e., γH2A, Exo1, RPA1, and RAD51, to monitor the HR pathway.

### DSBs induced by IR are efficiently repaired during the late S/G2 phases of the cell cycle

As cited above, the HR pathway acts primarily during the late S and G2 phases in model eukaryotes such as yeast and mammalian cells^38,39^. Therefore, following the dynamic recruitment of the HR pathway, we estimated the time required to repair DSBs in parasite cells. Considering that *T. brucei* lacks the canonical NHEJ pathway^15^, which occurs throughout the cell cycle and is a predominant mechanism of DSB repair in the G1 phase^38^, we studied the fate of cells in the G1 stage during IR treatment. Are parasites that have their DNA damaged in the G1 phase able to replicate until they reach the late S or G2 phases, where the HR pathway can perform repairs? Alternatively, is there a different pathway acting during the G1 or early S phases that prevents parasites from replicating?

**Figure 7:**
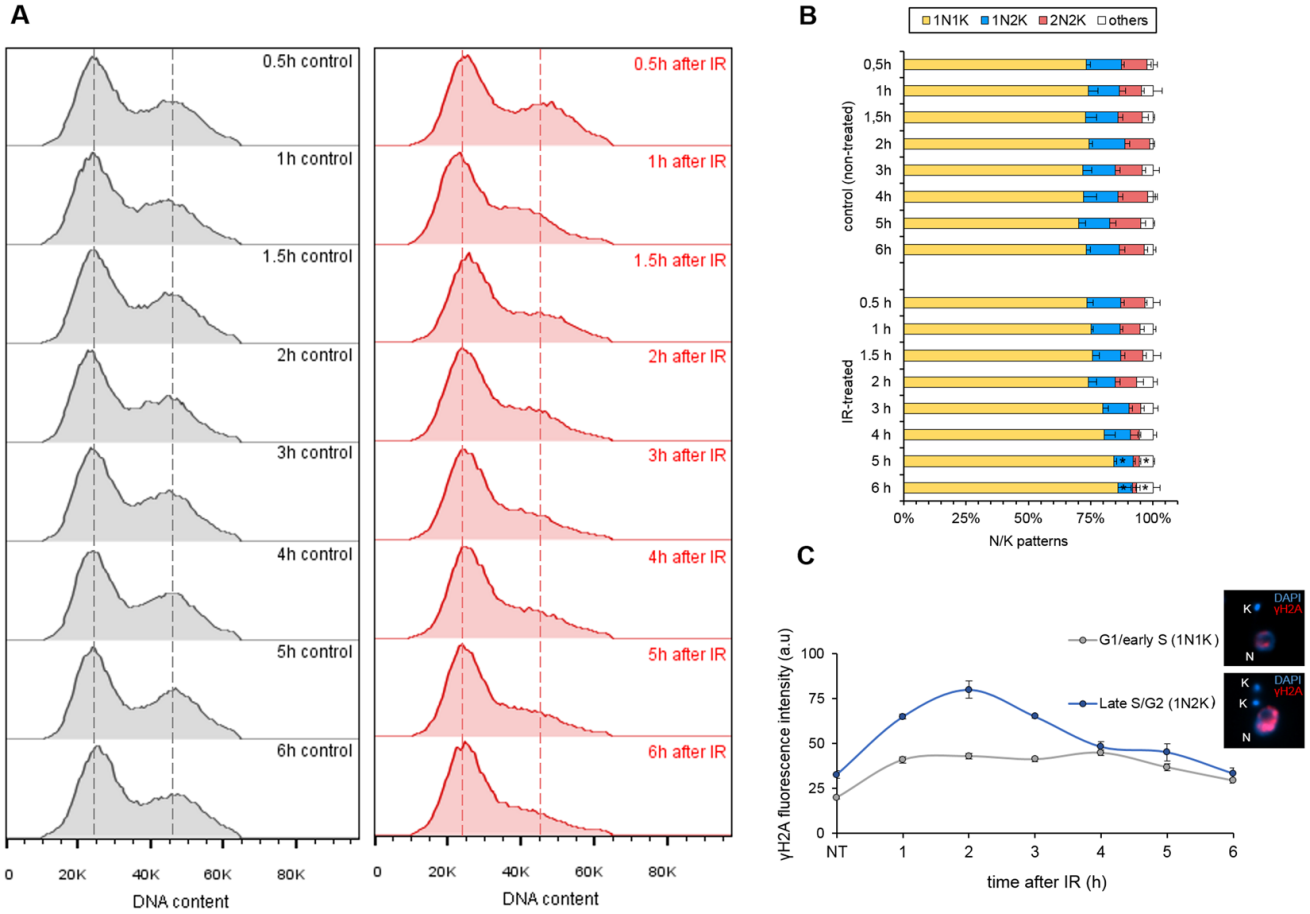
Effects of the IR response on the cell cycle. (**A**) DNA content analysis of PI-stained cells. The red histograms represent the IR-treated cells and the gray histograms represent the non-treated cells. This assay was performed in triplicate. Dotted lines represent the peak of fluorescence intensity of G1/S cells (left peak) and of G2/M cells (right peak). (**B)** A measurement of N/K patterns through the DAPI-staining of IR-treated and non-treated cells. The data represent the average of three independent experiments and the error bars represent the standard deviation. n > 100 for each sample. (**C**) Fluorescence intensity of γH2A, according to the cell cycle phases (G1/early S and late S/G2). Note that these cell cycle phases were estimated based on the duplication of the kinetoplast (K) and nucleus (N) during the cell cycle. The data represent the average of 50 analyzed cells from the G1/S phase and 15 analyzed cells from the late S/G2 phase.

To answer these questions, we analyzed the DNA content profile of the IR-treated parasites from 0 to 6 h after treatment and compared the results to non-treated parasites. The DNA content profiles revealed a gradual decrease in the prevalence of cells in the G2/M phase after IR treatment, with the lowest amount of cells in the G2/M phases occurring six hours after IR treatment (Fig. 7A). These data (which was collected in triplicate) corroborated the results that DNA damage is repaired by HR in the late S/G2 phases at approximately 5.5 h after IR exposure. In addition, we also observed parasites gradually accumulating in the G1/S phases beginning at 3 h after IR (Fig. 7A). We also evaluated the stages of the cell cycle after IR exposure by measuring the nucleus (N) and kinetoplast (K) patterns using DAPI-stained parasites (Fig. 7B). The result of this analysis showed an increase in the 1N1K population, followed by a decrease in the 1N2K and 2N2K populations from 2 to 6 h after IR treatment compared to the same pattern in non-treated cells (* represents p < 0.01 using Student’s t-test). This finding corroborates the DNA content analysis, because the 1N1K population represents cells in the G1/early S phases, and 1N2K/2N2K represents cells in the late S/G2, mitosis or cytokinesis phases. Notably, the ‘others’ pattern indicated in Fig. 7B represents multinucleated (cells with more than 2 nuclei) or aberrant cells (cells with 2N1K, 2N0K, 1N0K or 0N1K patterns). These results suggest that *T. brucei* procyclic forms have a repair pathway that detects DNA damage at the G1/S transition, which took more than 6 h to be finalized.

Finally, to demonstrate that the HR kinetics observed in Fig. 6 represent the process that occurs during the late S/G2 phase, we tested whether the phosphorylation and dephosphorylation of H2A occur in late S/G2. Therefore, we repeated the intensity analysis of the γH2A signal after IR, comparing the signal intensity of cells in G1/early S (1N1K pattern) with cells in late S/G2 (1N2K pattern). The results show that the predominant peak highlighted earlier for γH2A (Figs 2C and 6F) occurs predominantly in late S/G2 (Fig. 7C). Thus, we suggest that DSBs are repaired by HR in late S/G2. Moreover, a checkpoint response arrests cells at the G1/S transition phase, and we can speculate that if a DSB repair process occurs during the transition in the G1/S phase, then this process takes more than six hours and probably does not depend on high levels of γH2A.

**Figure 8:**
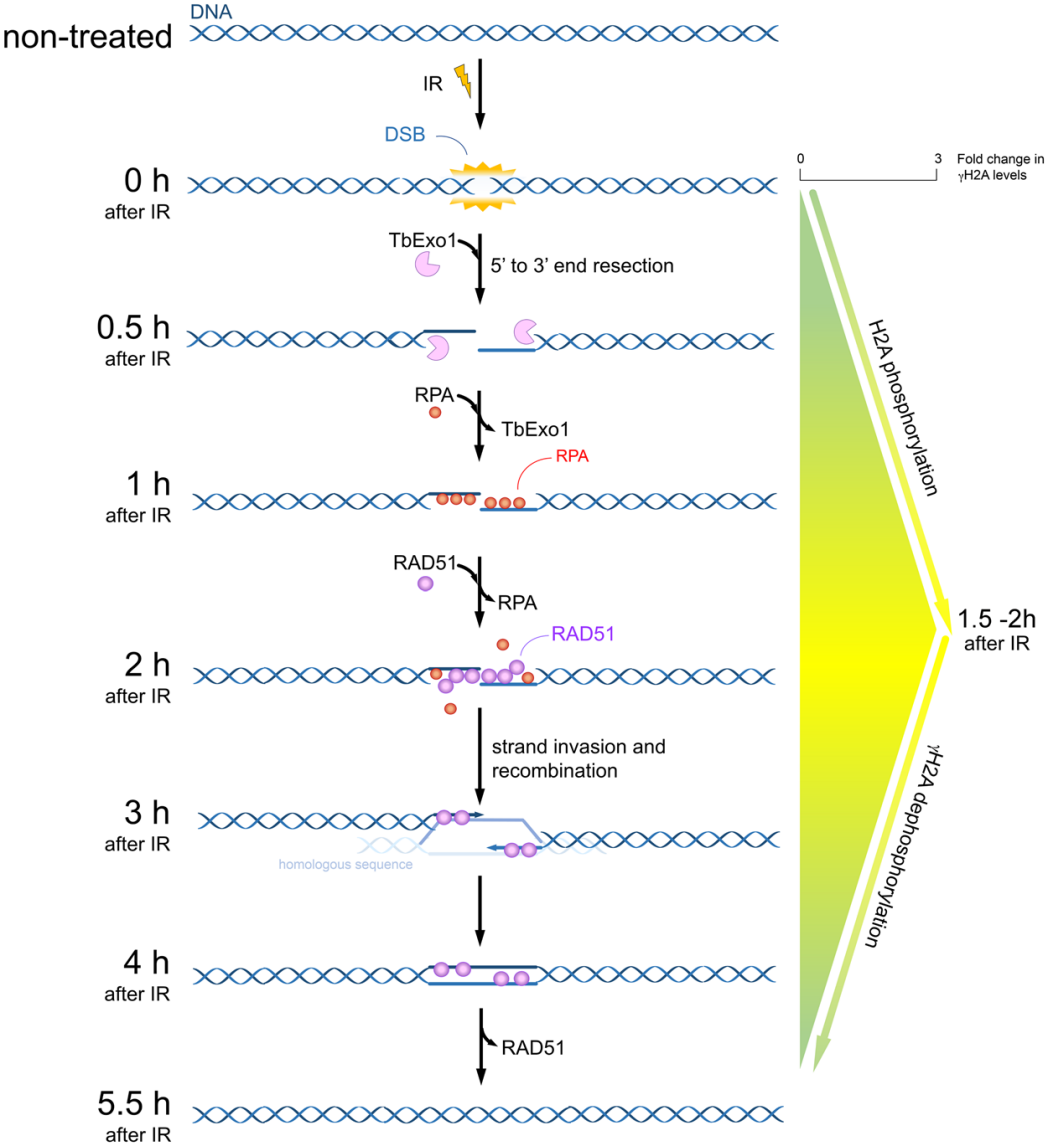
Model for the recruitment kinetics of HR repair proteins after IR. In *T. brucei* procyclic forms, IR exposure promotes the recruitment of the HR pathway onto DNA after DSB. The complete DNA repair process takes approximately 5.5 hours.

In Fig. 8, we propose a scheme for the HR recruitment kinetics in response to the DSBs generated by IR in *T. brucei* procyclic forms. In brief, the DSBs induced by 50 Gy IR led to a rapid recruitment of Exo1 at sites of DNA damage during the first half hour after IR treatment. The peak in DNA-bound RPA1 is achieved 1.5 h after IR, when the interaction between Exo1 and DNA decreases. From this period until two hours after IR, the presence of DNA-bound RPA decreases, while DNA-bound RAD51 increases. Additionally, the maximum H2A phosphorylation intensity is achieved 1.5 – two hours after IR. Together with the interactions between RAD51 and DNA (2–5 h after IR), γH2A dephosphorylation occurs. The complete process of DSB repair takes approximately 5.5 hours, as shown by the decrease in the γH2A levels and the minimal number of parasites in the G2/M phases at this time.

## Discussion

DSBs are one of the most lethal forms of DNA damage, and they must be repaired to maintain genomic integrity. To study DNA damage repair in procylic forms of *T. brucei*, we first evaluated the effects of different doses (50–175 Gy) of IR treatments on the cell culture growth and found that the 50 Gy dose affects parasite proliferation, impairing growth but enabling recovery after IR treatment (Fig. 1). Few studies have investigated IR treatments in *T. brucei*, and none have precisely addressed its radioresistance^40,41^. Our result shows that *T. brucei* procyclic radioresistance is lower than the resistance of *Leishmania amazonensis*^42^ and *T. cruzi*^43^, which have values of 500 Gy and 1500 Gy, respectively. The *T. brucei* procyclic radioresistance is closer to that of model eukaryotes, since the radioresistance of human embryonic stem cells^44^ and different human tumors range from 1 to 70 Gy^44,45^.

We then estimated the kinetics of DSB repair in *T. brucei* procyclic forms following IR treatment. The TUNEL assay and γH2A fluorescence intensity were used to monitor the appearance and disappearance of this type of DNA damage (Fig. 2). In mammalian cells, histone H2AX undergoes phosphorylation in response to DSBs^23–25^. However, H2AX has not been described in any trypanosomatids^35^ or yeast^46^. Instead, in these organisms, histone γH2A was identified as a candidate for playing the role of γH2AX, increasing the *in vivo* in response to DNA damage^35^. Our data showed that as in mammals^47^ and *Saccharomyces cerevisiae*^46^, *T. brucei* seems to exhibit a rapid phosphorylation of H2A after DSBs. TUNEL profiles were positive during the first 1.5 hours after IR, and the signal was negative after this period. However, the γH2A was significantly positive during the first six hours after DNA damage (Fig. 2). To explain this apparent discrepancy, we hypothesized that after IR, the rapid recruitment of the DNA repair machinery onto the DNA terminal ends prevents TdT enzymatic activity, making the TUNEL assay unable to measure the true intensity of DNA fragmentation after IR. We also observed basal levels of γH2A in non-treated (control) cells (Figs 2B and 6A). Basal levels of γH2A had not been previously reported in trypanosomatids^12,42,48^, except for in *T. brucei*^35^. Similar to *T. brucei* procyclic cells, unperturbed malignant human cells^49^ and mutant Chinese hamster ovary cells^50^ showed constitutive H2AX phosphorylation that varies according to the cell cycle phase. In these studies, cells in the S and G2/M phases exhibited greater levels of H2AX phosphorylation than cells in the G1 phase. These studies show that constitutive H2AX phosphorylation occurs in response to endogenous DSBs, which is probably generated as a result of DNA replication stress and/or the reactive oxygen species (ROS) generated throughout the cell cycle. The correlation between the presence of γH2A basal levels in non-treated *T. brucei* cells and DNA replication stress requires further investigation.

Considering that the presence of γH2AX on the chromatin may be enough to initiate the effective recruitment of the DSB repair machineries^51–53^, we are continuing our investigation of the possible kinetics of DSB repair in *T. brucei*. Because trypanosomatids apparently do not present a typical NHEJ due to the absence of the proteins DNA ligase IV and XRCC4^15^, we investigated the kinetics of recruitment for the primary proteins belonging to the HR pathway. In this way, we first analyzed the dynamics of TbRPA1, a protein that participates in the DNA damage repair machinery. Indirect immunofluorescence analysis revealed that TbRPA1 signals increased over the first half hour after DNA damage, and the maximum fluorescence intensity of TbRPA1 is observed one hour after IR treatment (Fig. 3A,B), primarily in cells in the late S/G2 phase (Fig. 3C). In addition to the TbRPA dynamics after DSBs, we also observed a double band of TbRPA1 in WB analysis from the total protein extracts at one hour after IR treatment (Figs 4A and 5C). A fractionated protein assay (Fig. 5A) revealed that the TbRPA1 upper band is the one that predominantly binds DNA in response to the DSBs generated by IR (Fig. 5C,D). We hypothesized that this upper band reflects a post-translational modification in TbRPA1. Evidence has shown that DNA repair proteins could be assembled and disassembled from complexes at DNA damage foci through the phosphorylation of RPA2^54^. However, no post-translational modifications were observed in RPA1 from different organisms in response to the DSB generated by IR. Additionally, studies have shown that the formation of protein complexes in response to DNA damage requires the interaction of multiple proteins with RPA1 through its N-terminal basic binding domain (DBD-F)^54^. Here, we show that, as suggested in *L. amazonensis*^16^, the natural absence of the N-terminal domain of TbRPA1 apparently did not affect the participation of RPA1 in signaling pathways involving DSB repair by the HR pathway and probably ATR kinase activation. The RPA1 signal shown in Fig. 6 probably corresponds to the DNA-bound RPA1, which we hypothesized could be the upper band presented in Fig. 4A. Thus, we ask why this upper band disappears after 1 h in Fig. 4. We suggest that due to differences in the exposure time, it is difficult to see the upper band of RPA1 in Fig. 4 since Fig. 4 shows an extract of the total proteins.

To continue elucidating the recruitment kinetics of the HR machinery in *T. brucei*, we evaluated the time course of the DNA-bound repair proteins in the HR pathway besides RPA1, namely, TbExo1, which processes the DNA ends, and TbRAD51, which is involved in pre-synaptic complex formation and in searching for homologous sequences. DNA-bound TbExo1 rapidly reaches a peak of intensity before γH2A and TbRPA1 (Fig. 6). We do not have enough data to speculate about the role of ATM or ATR in γH2A phosphorylation. However, we would expect a faster phosphorylation of H2A if it were phosphorylated by ATM or DNA-PK. Thus, the observed peak in the DNA-bound TbExo1 intensity precedes the question as to whether the γH2A peak makes sense if we consider that the oligonucleotides generated from exonuclease activity on DSBs stimulate ATM autophosphorylation. This finding probably increases the γH2A signals at the beginning of the response, which occurs with model eukaryotes^18^. Alternatively, we consider that part of the H2A phosphorylation is performed by ATR, which is recruited by the complex RPA-ATRIP, although there is no description of ATRIP in trypanosomatids^55^. In any case, the rapid recruitment of TbExo1 is necessary to process the DSBs induced by IR, generating ssDNA segments that will be bound by TbRPA1, in addition to stimulating an increase in the γH2A signal. This coordinated recruitment process leads to a maximum intensity of TbRAD51 2 h after IR, when the γH2A signals begins to decrease. The maximum intensity of DNA-bound TbRAD51 is probably activated as a negative feedback for the beginning of γH2A dephosphorylation, restoring the normal basal condition five hours after DSBs generated by IR^56,57^.

Finally, we performed a DNA content analysis to investigate whether HR repair occurred preferentially during the late S and G2 phases (Fig. 7A,B). Interestingly, we observed an unexpected absence of arrest in the late S/G2 phase (Fig. 7A,B), a small arrest in the G1 phase in the first hours after IR, and a progressive decrease in G2/M that seems to be more evident 5–6 h after IR treatment (Fig. 7A,B). In addition, the measurement of N/K patterns indicated an increase in the 1N1K population, followed by a decrease in the 1N2K and 2N2K populations from 2 to 6 h after IR exposure (Fig. 7B). These data corroborate the DNA content analysis. Moreover, a predominant fluorescence peak for γH2A that was primarily observed in late S/G2 (Fig. 7C) complements both the DNA content and the N/K pattern results (Fig. 7). The probable absence of a mitosis/cytokinesis checkpoint in *T. brucei* procyclic cells^58^ might contribute to the profile observed during DNA content analysis. In other words, if a DNA-damaged cell is not repaired during late S/G2, it will probably migrate to the next checkpoint, which is located in the G1/S transition. We believe that this G1/S arrest presented in *T. brucei* procyclic cells after IR exposure is due to the cell’s attempt to repair DSBs via the MMEJ pathway (an alternative NHEJ pathway). This hypothesis is sustained by a study in *T. brucei* reporting that the DNA end joining repair of linear molecules is independent of the Ku heterodimer, indicating that this repair mechanism is distinct from the NHEJ and is guided by the sequence microhomology repair pathway^15^, which is subsequently described as MMEJ in trypanosomatids^9,12,14^. It is important to note that we cannot make any correlations between our data (which were collected using procyclic forms) and the mechanism of variant surface glycoprotein (VSG) switching performed by bloodstream forms of *T. brucei*, because VSGs are located in specific genetic clusters (known as the BES, or bloodstream expression site) that are only transcribed in the bloodstream forms of *T. brucei*^59,60^.

Taken together, these results suggest that procyclic *T. brucei* parasites have a highly efficient HR pathway for repairing DSB, apparently bypassing the typical G2/M cell cycle arrest when they are challenged by mild IR exposure. Moreover, the observation of G1/S arrest probably indicates that DSB repair also occurs via another pathway, which is less efficient than HR, which we believe to be MMEJ. In conclusion, Fig. 8 presents the order of recruitment for the primary proteins involved in the HR pathway in response to DSB in procyclic forms of *T. brucei*. In addition to proposing the recruitment kinetics for key HR players in response to DSBs for the first time in this organism, this study also provides potential targets for the development of antiparasitic therapy, an extremely important step towards the eradication of this parasite, which afflicts millions of people around the world.

## Methods

### Ionizing radiation treatment and cell viability assay

*Trypanosoma brucei* (Lister strain 427) procyclic forms were cultured at 28 °C in SDM79 medium supplemented with 10% (v/v) fetal bovine serum. Exponentially growing parasites were subjected to different doses of ionizing radiation (IR) (50, 100, 150, and 175 Gy) from a Gamma Cell 220 cobalt 60 irradiator unit with a rate dose of 913 Gy/h.

### Fluorometric TUNEL assay

Irradiated parasites (treated with 50 Gy) were harvested by centrifugation (∼5·10^7^ parasites) at 1700 g for 5 min and washed twice in 1X PBS (137 mM NaCl, 2.7 mM KCl, 10 mM Na_2_HPO_4_, and 2 mM KH_2_PO_4_ pH 7.4). The parasites were then fixed for 20 min with 1% methanol diluted in cold 1X PBS (1 mL), which was added by dripping the 1X PBS with methanol onto the parasites under gentle homogenization. These parasites were incubated overnight at 4 °C in a fresh 1 mL solution of 70% ethanol diluted in PBS. The DeadEnd™ Fluorometric TUNEL System kit (Promega) was used according to the manufacturer’s instructions to detect DNA fragmentation. Reaction controls were included; a negative control (C−) was tested without the rTdT enzyme; the positive control (C+) employed parasites that were pre-treated with DNase I (ThermoFisher) for 10 min. After incubating, the parasites were pelleted at 2600 g for 10 min and then homogenized for 10 min in PBS containing 0.1% Triton X-100 and 3% BSA in PBS. The samples were then prepared for fluorescence microscopy and flow cytometry analysis. Positive fluorescence profiles from 10,000 parasites were detected using the BL1A channel of the flow cytometer (NxT Attune – Thermo Scientific).

### Indirect Immunofluorescence assay (IIF)

Irradiated parasites (50 Gy) or control cells were harvested by centrifugation (∼5.10^6^ parasites) at 1700 g for 5 min and washed twice with 1X PBS. The parasites were then fixed for 15 min using 4% paraformaldehyde under gentle agitation. Washed parasites were homogenized in 1X PBS and added to Teflon-coated microscope slides (Tekdon) for 15 min. The parasites were then washed three times on slides for two minutes each time with blocking solution (3% BSA in 1X PBS) and permeabilized for 10 min with 0.1% Triton X-100 diluted in 1X PBS. The washed parasites were then incubated at room temperature for 2 h in an antisera solution containing the anti-γH2A antibody^35^ (kindly provided by the Wellcome Centre for Molecular Parasitology, University of Glasgow) diluted at 1/1000 in 1% BSA in 1X PBS or anti-RPA1^37^, which was produced in our lab, and diluted at 1/1000 in 1% BSA in 1X PBS. The parasites were then washed three times on slides and incubated with blocking solution for 20 min. These washed parasites were then incubated for 1 h with an Alexa Fluor 555 labeled goat anti-rabbit IgG secondary antibody (Thermo Scientific) that was diluted at 1/500 in 1% BSA in 1X PBS. Afterwards, the parasites were washed five times, and Vectashield Mounting Medium (Vector) containing 4′,6-diamidino-2-phenylindole dihydrochloride (DAPI) was used as an anti-fade mounting solution and to stain the nuclear and kinetoplast DNA. Images were acquired using an Olympus Bx51 fluorescence microscope (100× oil objective) attached to an EXFO Xcite series 120Q lamp and a digital Olympus XM10 camera with Olympus Cell F camera controller software. Image capture conditions were set using non-treated cells. The intensity of cell staining was estimated based on 100 cells per sample using Olympus Cell F tools. A mean comparison test was performed using The R Project for Statistical Computing software – R. When necessary, images were merged using Olympus Cell F software.

### Flow cytometry analysis

Irradiated parasites (50 Gy) were harvested by centrifugation (∼8.10^6^ parasites) at 1700 g for 5 min and washed one time in 1X PBS. The parasites were then fixed for 15 min with 4% paraformaldehyde in 1X PBS under gentle agitation. Afterward, they were washed with 1% BSA in 1X PBS and permeabilized for 15 min with 1% saponin in 1% BSA diluted in 1X PBS. The parasites were then washed again and incubated for 45 min at 37 °C with propidium iodide (PI) solution in 1X PBS containing 10 µg/mL PI and 20 µg/mL RNase. The DNA content from 20,000 events was analyzed using a flow cytometer (NxT Attune – Thermo Scientific). The channel gain conditions were set based on non-treated cells as follows: FSC (320), SSC (360), BL1 (400), BL2 (620), and BL3 (540).

### Protein expression, purification and *Tb*Exo1 antisera production

To produce the recombinant protein TbExo1, the protein coding sequence (accession number: Tb427.05.2450-t26_1, Molecular Weight: 115876, http://tritrypdb.org/tritrypdb/) was amplified by PCR from *T. brucei* genomic DNA using specific primers with restriction sites for the NheI and NotI enzymes (GCTAGCATGGGCGTGCCAAAGTTC and GCGGCCGCTCAAATTTGTAACTTCACC). The amplified fragments were inserted into the pJET1.2/blunt (Thermo Scientific) cloning vector and transformed into *E. coli* XL1 blue cells. Next, the TbExo1 coding sequences were removed from the pJET1.2/blunt vector, inserted into pET-28a (+) with a 6XHis-tag to facilitate protein purification and transformed into *E. coli* BL21 Rosetta cells. Protein expression was induced using 1 mM isopropyl thio-β-d-galactopyranoside (IPTG) at 37 °C for 4 h (see Supplementary Fig. 3A). The cells were harvested by centrifugation (3200 g, 10 min, 4 °C) and suspended in lysis buffer (50 mM Tris-HCl pH 8.0, 300 mM NaCl, 1% N-lauroyl sarcosine, 1 mM PMSF, 1 mM dithiothreitol (DTT) and 1× protease inhibitor cocktail (Roche)). The cells were then disrupted by cold sonication followed by centrifugation (9000 g, 20 min, 4 °C). The proteins in the soluble fraction were loaded into a Nickel Sepharose column (Ni-NTA, Qiagen) that was previously equilibrated with starting buffer (50 mM Tris-HCl pH 7.5, and 0.3 M NaCl). The proteins that were bound to the column were eluted with equilibration buffer (50 mM Tris-HCl and 300 mM NaCl) in a linear pH gradient (from pH 10 to pH 8, 5, and 3) (see Supplementary Fig. 3B). The eluted samples were concentrated using an Amicon® Ultra-4 Centrifugal Filter Units (Millipore), recovered in starting buffer (50 mM Tris-HCl pH 7.5 and 0.3 M NaCl) and separated by 6% SDS-PAGE electrophoresis. rTbExo1 was used to generate TbExo1-specific antisera (Proteimax, São Paulo) (see Supplementary Fig. 3C).

### Total and fractionated cellular protein assay

Irradiated parasites (50 Gy) were harvested by centrifugation (∼1.10^8^ parasites) at 1700 g for 5 min and washed twice in 1X PBS. The total protein extract samples were then obtained by treating the parasites with 2× reducing sample buffer containing 1 M Tris pH 7.0, 20% SDS, 5% glycerol, 0.1% bromophenol blue, and 5% β-mercaptoethanol. To obtain fractionated protein extracts, fresh samples were solubilized for 10 min at 4 °C under gentle agitation in extraction buffer containing 10 mM Tris-HCl pH 7.4, 100 mM NaCl, 300 mM sucrose, 3 mM MgCl_2_, 50 mM NaF, 1 mM Na_3_VO_4_, 0.5 mM PMSF, 0.1% Triton X-100, and EDTA-free protease inhibitor cocktail (Sigma Aldrich). The samples were then pelleted (2550 g, 2 min, 4 °C), and the supernatants were saved as the soluble fraction (soluble fraction I). The pellets were treated with extraction buffer again, and the second round of supernatants was saved as the soluble fraction (soluble fraction II). The pellets were then treated for 30 min at 37 °C with 350U DNaseI (ThermoFisher) diluted in sterile water. The samples were then pelleted, and the supernatants were saved as the DNase-released fraction (DNase released fraction I). The samples were then boiled for five minutes at 95 °C, fractionated by SDS-PAGE (30 µL per line) and transferred electrophoretically to a nitrocellulose membrane (GE Life Science). After blocking overnight in 1X Tris-buffered saline (1X TBS) containing 5% non-fat dry milk, the blots were washed with 1X TBS 0.05% Tween20 five times for five minutes each. After being washed, the membranes were cropped and incubated at room temperature under gentle agitation for four hours with the appropriate antisera solution in 1X TBS 3% non-fat dry milk-containing anti-γH2A antibody (1/2,000)^35^ and anti-RPA1 antibody (1/1,000) produced in our lab^37^, an anti-EXO1 antibody (1/5,000) and anti-RAD51 antibody (1/500)^30^ (which was kindly provided by the Wellcome Centre for Molecular Parasitology, University of Glasgow), and anti-HSP70 antibody (1/10,000) (Abcam) and anti-GAPDH antibody (1/3,000)^61^ (kindly provided by the Laboratory of Biochemistry of Tryps - LaBTryps, University of São Paulo) or H3 antibody (1/3,000) (Abcam). After being washed, the blots were incubated with a horseradish peroxidase (HRP)-conjugated secondary antibody for one hour at room temperature. Following additional washes, antibody binding was detected with the Immobilon Western Chemiluminescent HRP Substrate (Millipore). For the quantitative analysis of protein bands from immunoblotting experiments, digital images of the membranes were acquired using the chemiluminescence and fluorescence imaging system UVITEC (UVITEC Cambridge), and the band optical densities were quantified using ImageJ software (National Institutes of Health). To account for possible variations in sample loading, HSP70 or GAPDH bands were analyzed for soluble proteins, and H3 bands for fractionated proteins were analyzed in a similar fashion. All the protein bands were quantified, normalization included, by comparing them to the respective soluble or chromatin-bound controls.

## Electronic supplementary material

Supplementary Information 1, 2 and 3
