# Supplementary material for: Recruitment kinetics of the homologous recombination pathway in procyclic forms of Trypanosoma brucei after ionizing radiation treatment
Source: Sci Rep. 2018 Mar 29;8:5405. doi: 10.1038/s41598-018-23731-6 (PMC5876374; doi:10.1038/s41598-018-23731-6)
Supplement: Supplementary file 1 — Supplementary Information 1, 2 and 3 [file 41598_2018_23731_MOESM1_ESM.pdf]

Recruitment kinetics of homologous recombination pathway in procyclic forms of *Trypanosoma brucei* after ionizing radiation treatment

Paula Andrea Marin[1], Marcelo Santos da Silva[1], Raphael Souza Pavani[1], Carlos Renato Machado[2], and Maria Carolina Elias[1,*]

[1] Cell Cycle Laboratory (LECC) - Center of Toxins, Immune Response and Cell Signaling (CeTICS), Butantan Institute, São Paulo, São Paulo, 05503-900, Brazil. [2] Biochemical and Immunology Department, Institute of Biomedical Science, ICB, Federal University of Minas Gerais (UFMG), Minas Gerais, Belo Horizonte, 31270-901, Brazil.

* Corresponding author: carolina.eliassabbaga@butantan.gov.br

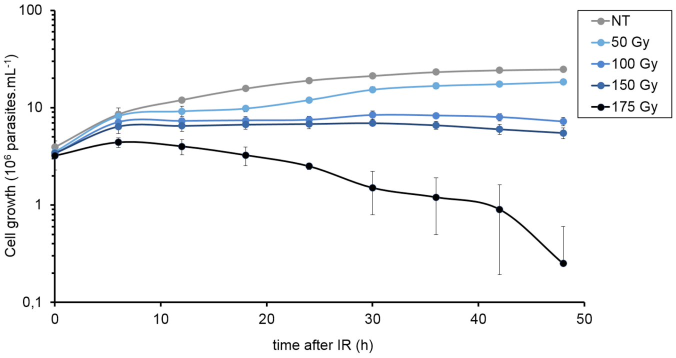

Figure 1. Cell reversibility afterIR treatment. The graph shows the cell growth every 12 h up to 48 h after being exposed to different doses of IR. The data represent the average of three independent experiments, and the error bars represent the standard deviations.

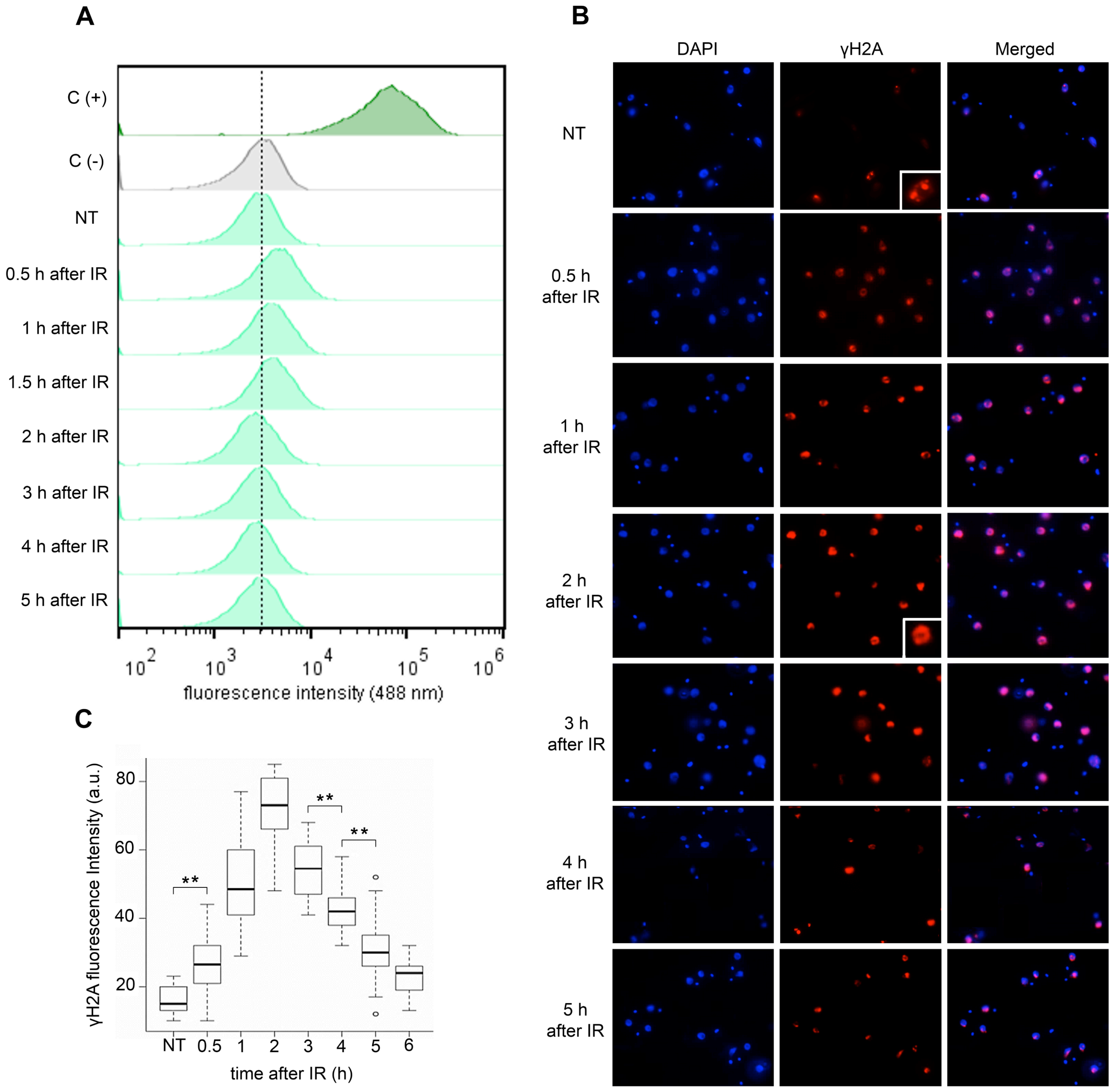

Figure 2. DNA fragmentation detection and H2A phosphorylation after IR. The parasites were treated with 50 Gy of IR and **(A)** the DNA fragmentation was measured using terminal deoxynucleotidyl transferase (TdT-TUNEL) after treatment. C(-) represents a negative control in which the TdT enzyme was not present. In C(+) the samples were pre-treated with DNase I generating therefore the maximum positive fluorescence intensity signal.. Dotted line was used to indicate the peak that means negative fluorescence. Therefore, peak to the right of dotted line was considered positive. **B)** The cells were analyzed by IIF to check the γH2A fluorescence intensity after IR treatment. **C)** Box plots represent the measurement of the γH2A fluorescence intensity in the IIF assay. The data represent the average for 100 analyzed cells. Bars represent the standard deviation. (**) Indicate significant differences compared to the non-treated cells via a Friedman and Wilcoxon test.

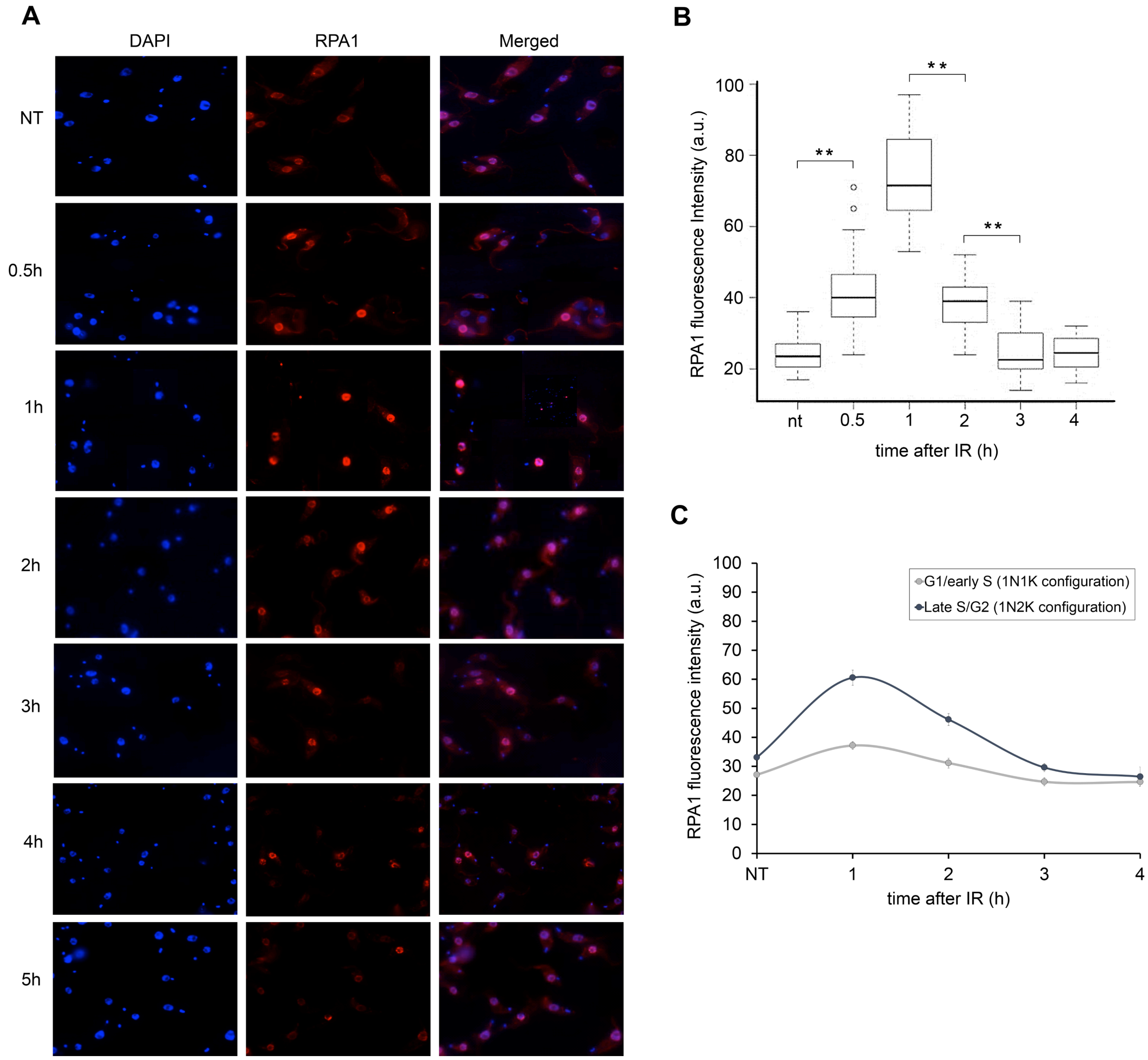

Figure 3. Replication protein A (RPA1) expression increases after IR. The parasites were treated with 50 Gy of IR. **A)** Immunofluorescence using anti-RPA1 at different time points after treatment. B) Box plots representing the RPA1 fluorescence intensity of the IIF assay presented in A. The data represent the averages of 100 analyzed cells, and the bars represent the standard deviation. (**) Indicate significant differences compared to the non-treated cells as determined by the Friedman and Wilcoxon tests. **C)** Fluorescence intensity of RPA1, according to the cell cycle phase (G1/early S and late S/G2). The cell cycle phases were estimated based on the duplication of the kinetoplast (K) and nucleus (N) during the cell cycle[62,63]. Data represent the average of 50 analyzed cells for the G1/S pattern and 15 analyzed cells for the late S/G2 pattern.

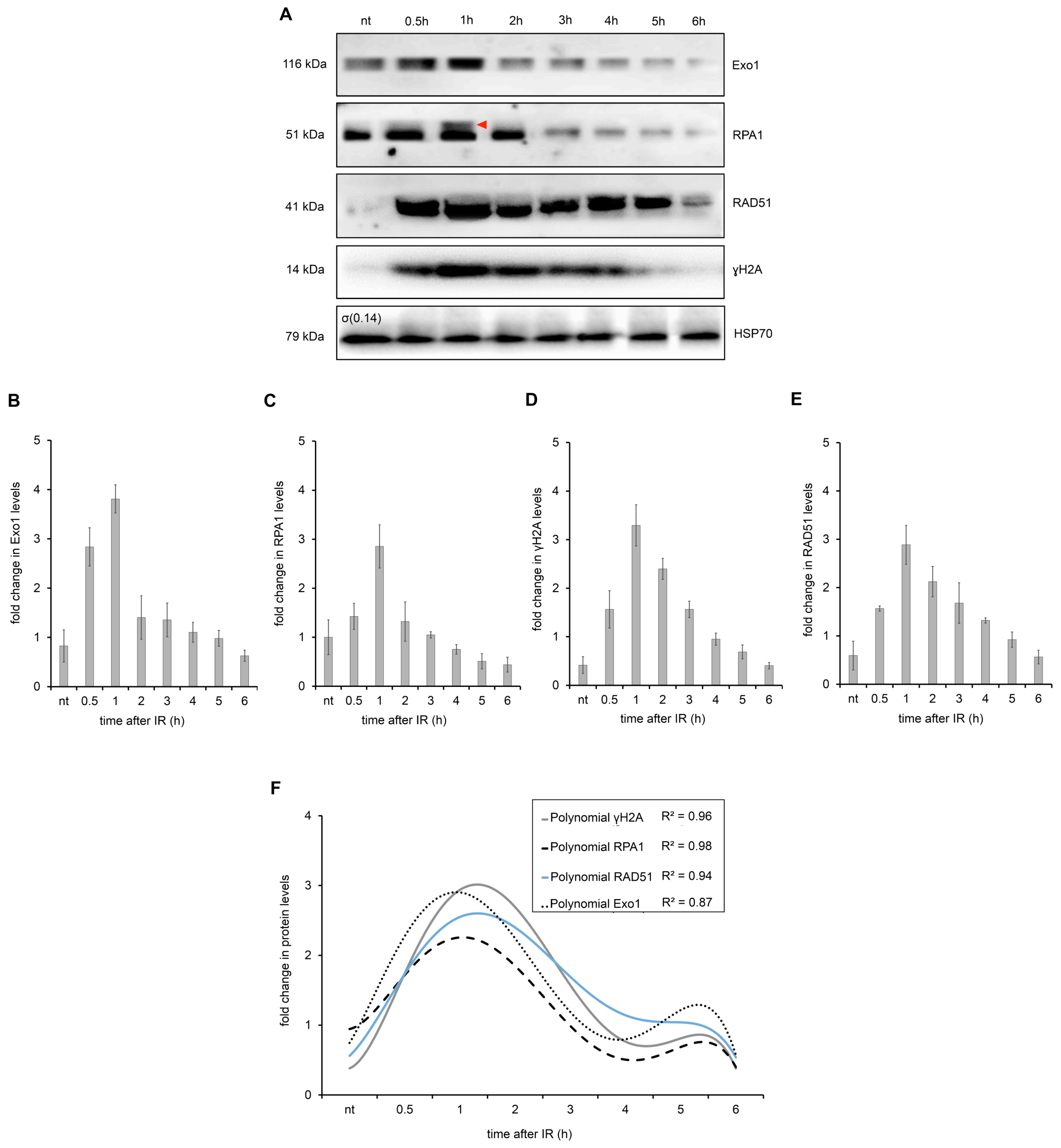

Figure 4. Homologous Recombination (HR) repair proteins in total protein extract after IR. **A)** WB analysis of the total protein extract after 50 Gy of IR exposure. HSP70 protein was used as an internal control. The red triangle on the RPA1 WB represents a possible RPA1 protein modification. **B-E)** A quantification of the relative intensity of Exo1 **(B)**, RPA1 **(C)**, γH2A **(D)** and RAD51 **(E)** protein after IR treatment. The data represent the averages of three independent experiments, and error bars represent the standard deviation. **F)** The sixth degree polynomial regression of WB quantifications. Non-parametric estimation curves and the R2 factor are presented. Notably, the Western blot membranes were cropped to avoid cross-reactions between secondary antibodies during the revealing process.

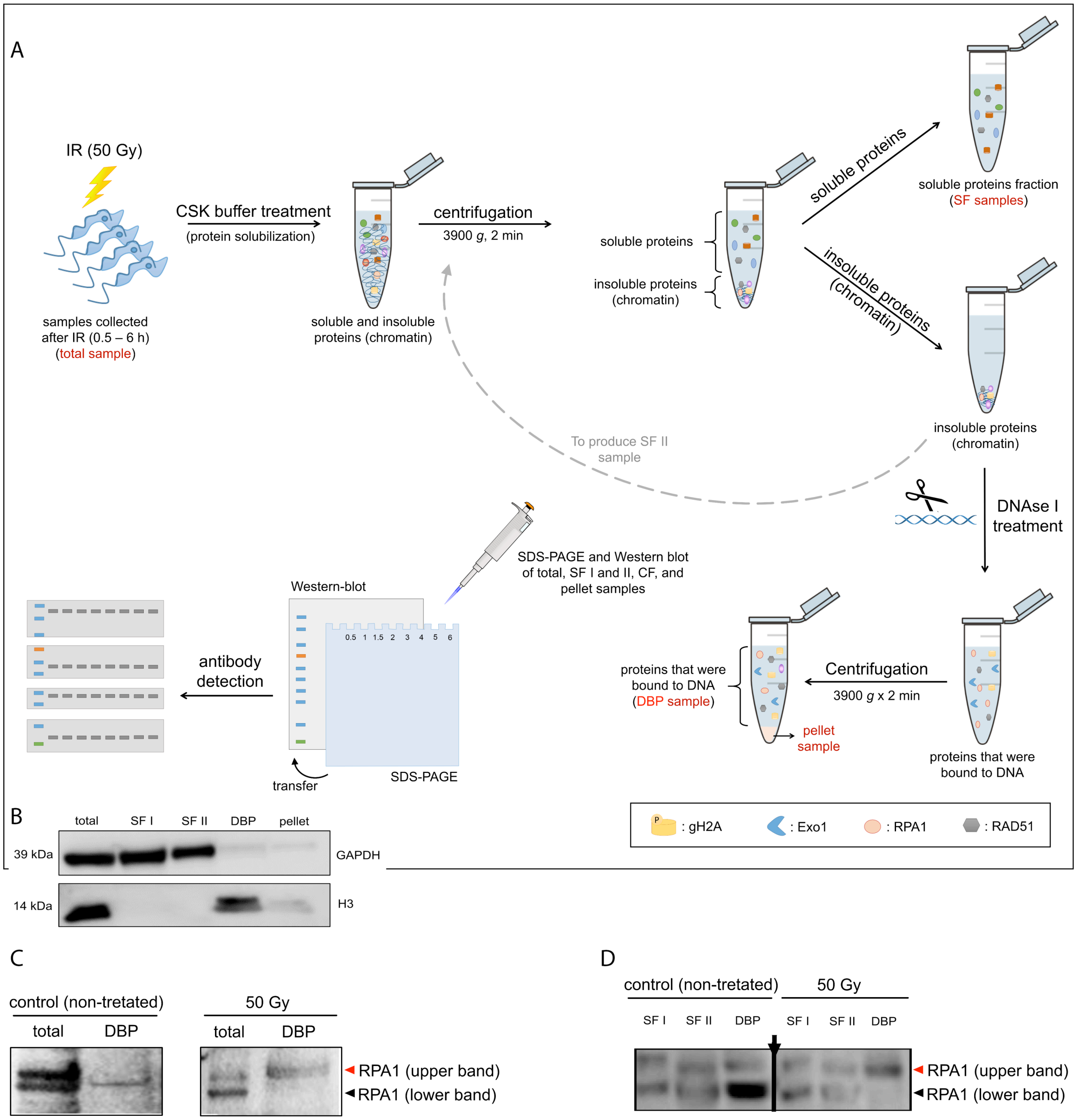

Figure 5. Fractionation assay and WB analysis of RPA1 bands after IR. A) Scheme showing the primary steps of the fractionation assay. The names indicated in red represent the fractions used in WB analysis**. B)** The GAPDH protein was used as an internal control for the soluble protein fraction (SFI and II). The H3 protein was used as an internal control for the DNA-bound protein fraction (DBP). Western blot membranes were cropped to avoid cross-reactions between secondary antibodies during the revealing process. **C and D)** Molecular weight migration profile of RPA1 in total, soluble protein fraction (SFI and II) and DNA bound protein (DBP) extracts after IR treatment. Arrow in panel **D** indicates a region where a lane was excluded from the gel, but all samples were analyzed in an unique gel.

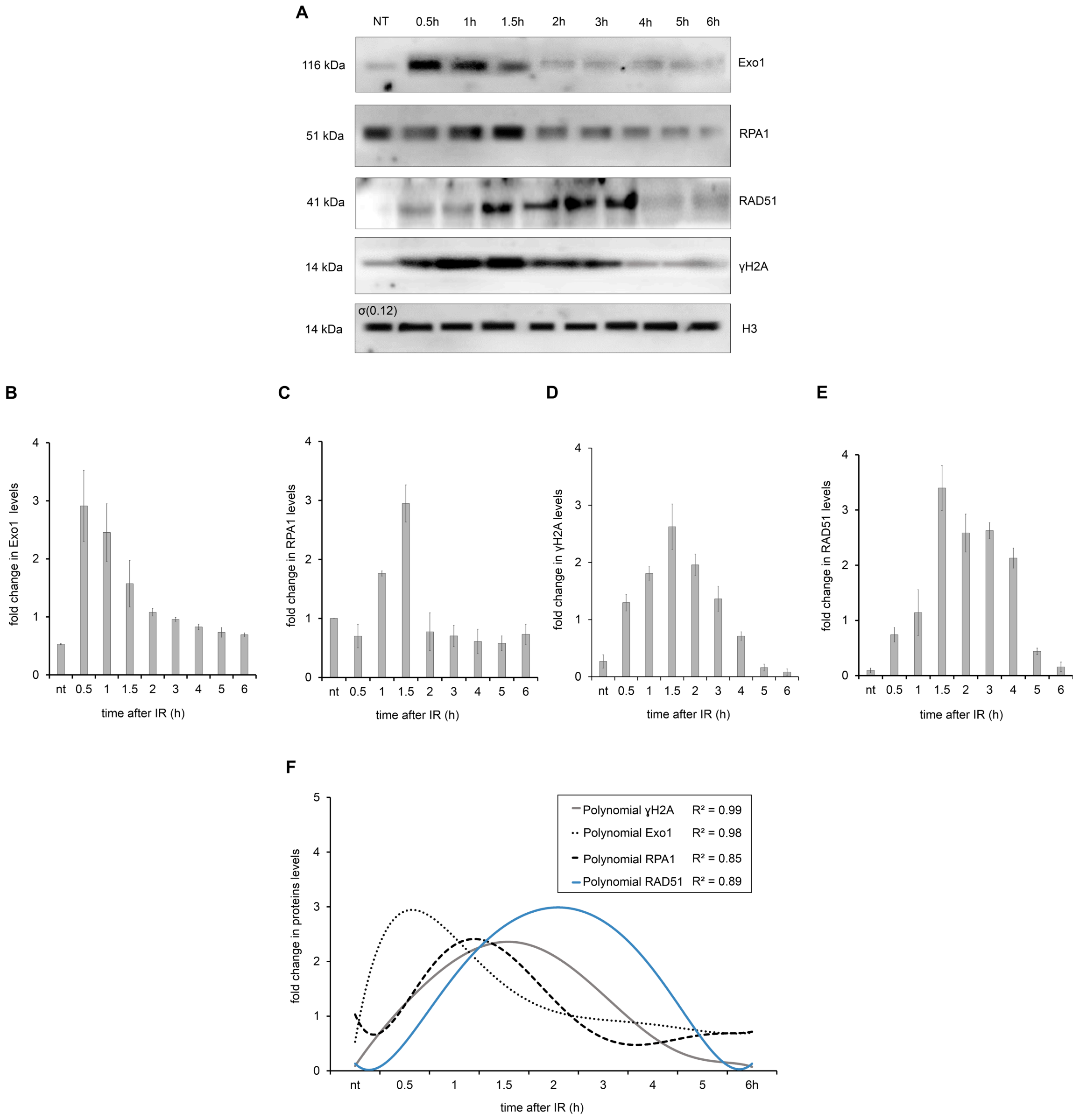

Figure 6. Recruitment of Homologous Recombination (HR) repair proteins to DNA after IR. A) WB analysis of DNA-bound proteins (DBP) derived from the fractionation assay after IR treatment. The H3 protein was used as an internal control. **B)** A WB quantification of the Exo1 relative intensity after IR treatment. **C)** A WB quantification of the RPA1 relative intensity after IR treatment. **D)** A WB quantification of the γH2A relative intensity after IR treatment. **E)** WB quantification of the RAD51 relative intensity after IR treatment. The data represent the average of three independent experiments and the error bars represent the standard deviations. **F)** A sixth-degree polynomial regression of the WB quantifications. Non-parametric estimation curves and R2 factors are presented. Notably, the Western blot membranes were cropped to avoid cross-reactions between secondary antibodies during the revealing process.

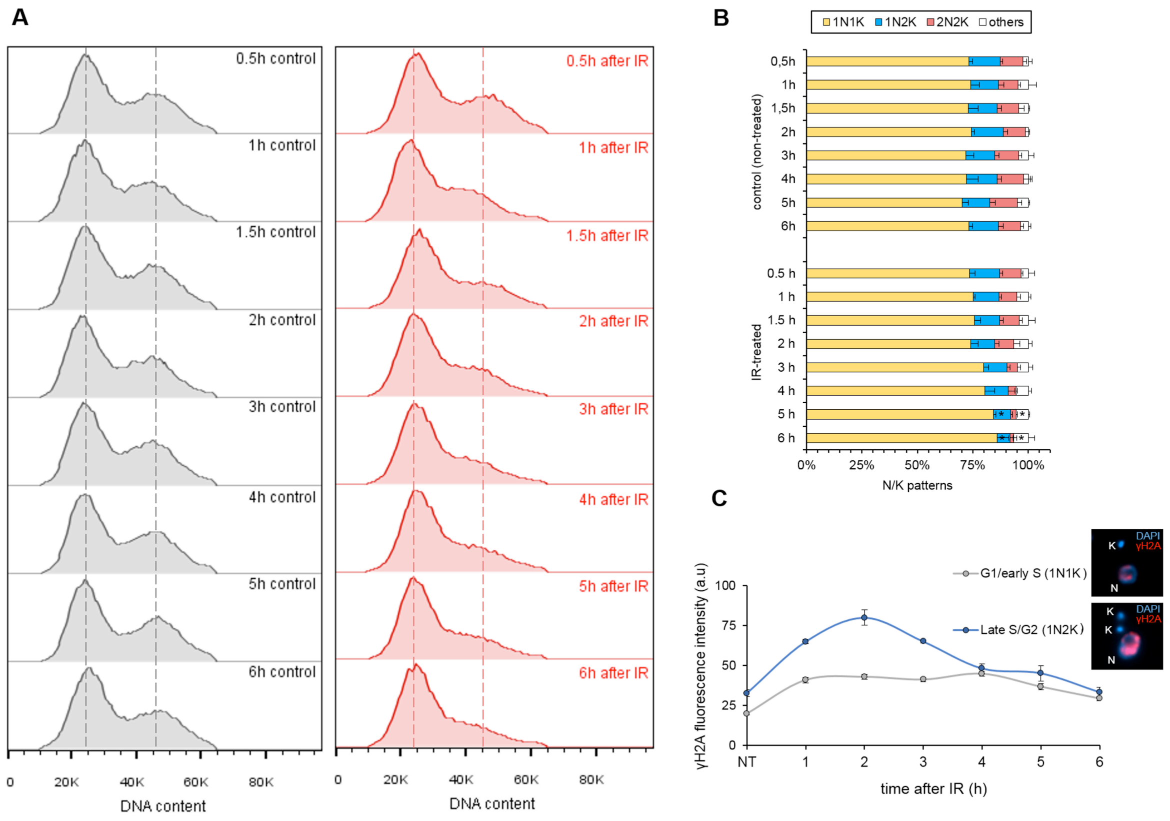

Figure 7. Effects of the IR response on the cell cycle. **A)** DNA content analysis of PI-stained cells. The red histograms represent the IR-treated cells and the gray histograms represent the non-treated cells. This assay was performed in triplicate. Dotted lines represent the peak of fluorescence intensity of G1/S cells (left peak) and of G2/M cells (right peak). **B)** A measurement of N/K patterns through the DAPI-staining of IR-treated and non-treated cells. The data represent the average of three independent experiments and the error bars represent the standard deviation. n > 100 for each sample. **C)** Fluorescence intensity of γH2A, according to the cell cycle phases (G1/early S and late S/G2). Note that these cell cycle phases were estimated based on the duplication of the kinetoplast (K) and nucleus (N) during the cell cycle. The data represent the average of 50 analyzed cells from the G1/S phase and 15 analyzed cells from the late S/G2 phase.

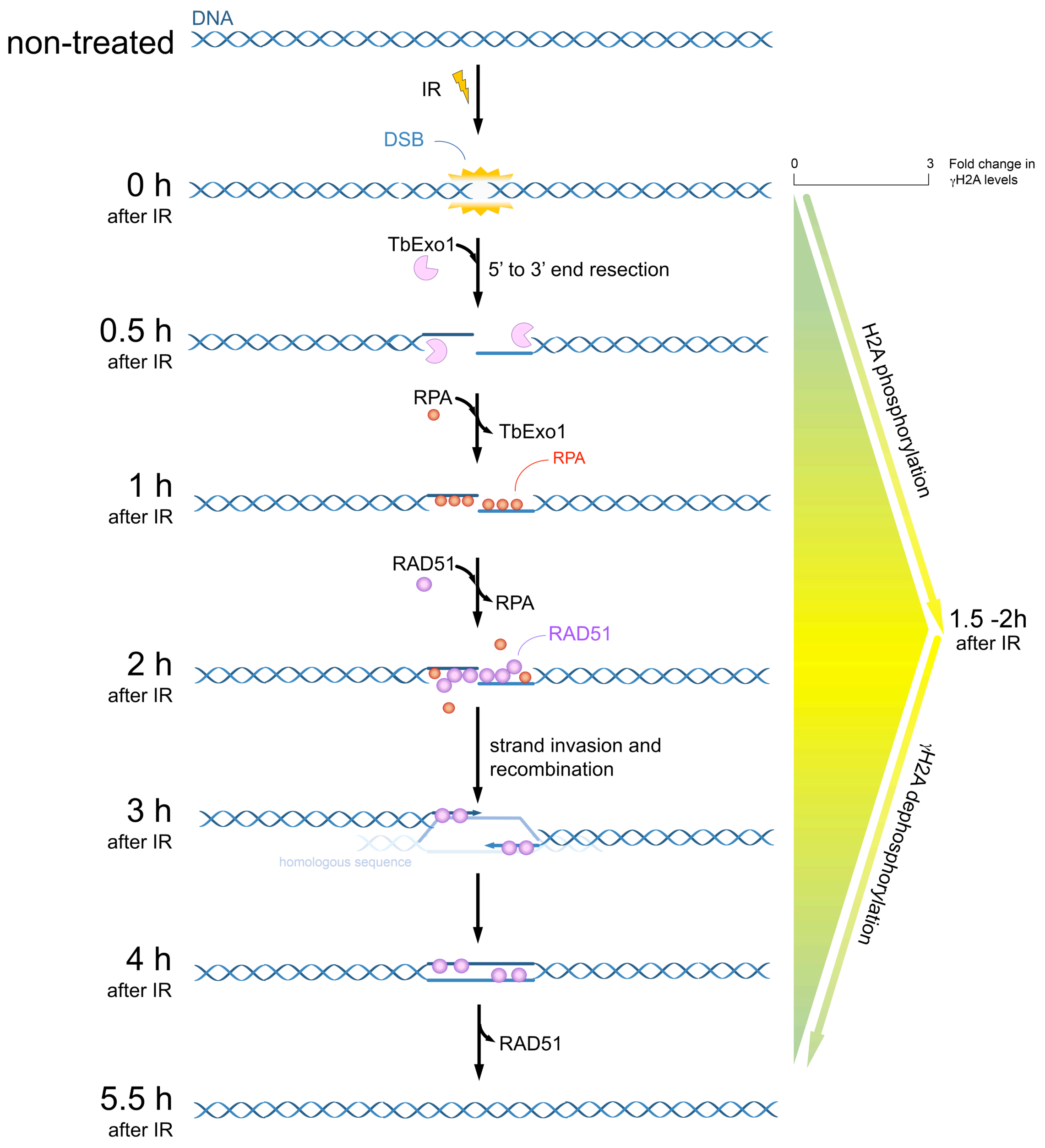

Figure 8. Model for the recruitment kinetics of HR repair proteins after IR. In *T. brucei* procyclic forms, IR exposure promotes the recruitment of the HR pathway onto DNA after DSB. The complete DNA repair process takes approximately 5.5 hours.

Supplementary information

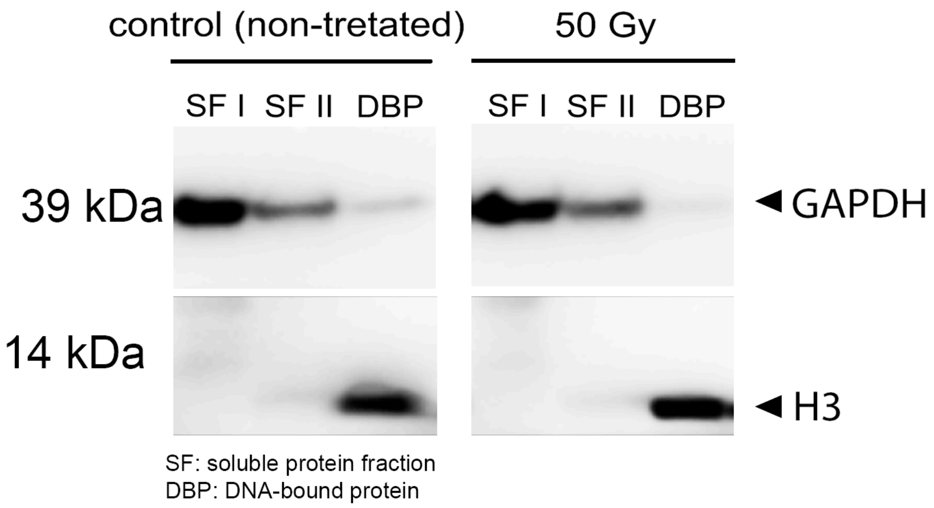

Supplementary Figure 1. Control of fractionated extraction of soluble and DNA bound proteins. Cells were exposed to IR and treated with lyses buffer in order to obtain soluble proteins (soluble fraction – SF). After a second round of treatment with lyses buffer, pellets were digested with DNAse in order to get DNA bound proteins (DBP). Samples were then submitted to SDS-PAGE, transferred to nitrocellulose membrane and incubated with anti-GAPDH and with anti-histone H3.

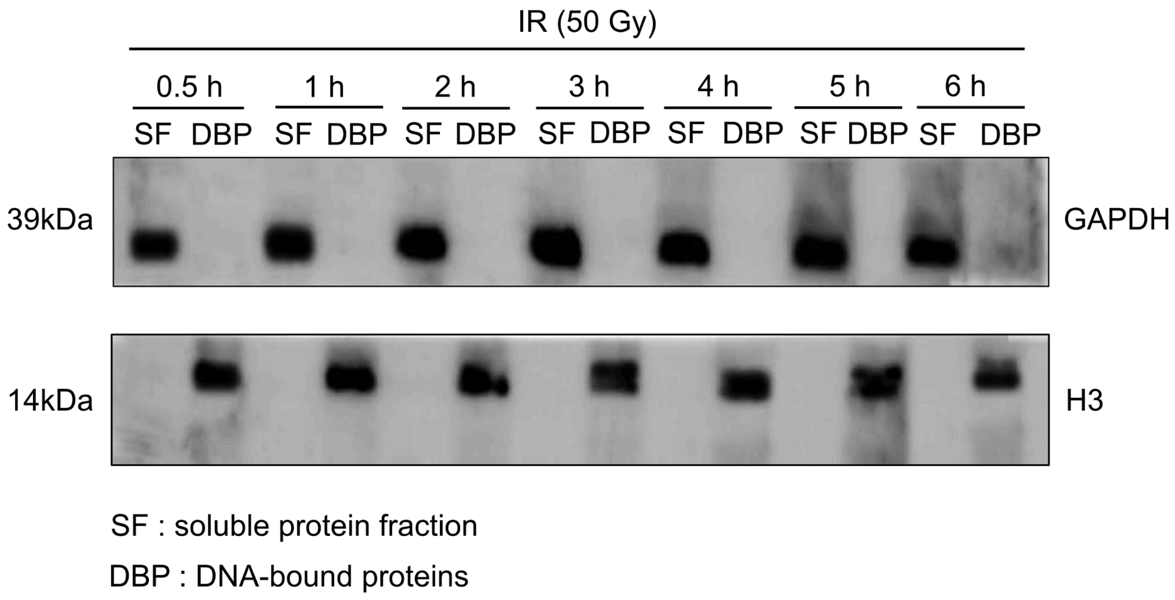

Supplementary Figure 2. Control of fractionated extraction of soluble and DNA bound proteins. Cells were exposed to IR and different times of exposition cells were treated with lyses buffer in order to obtain soluble proteins (soluble fraction – SF). After a second round of treatment with lyses buffer, pellets were digested with DNAse in order to get DNA bound proteins (DBP). Samples were then submitted to SDS-PAGE, transferred to nitrocellulose membrane and incubated with anti-GAPDH and with anti-histone H3.

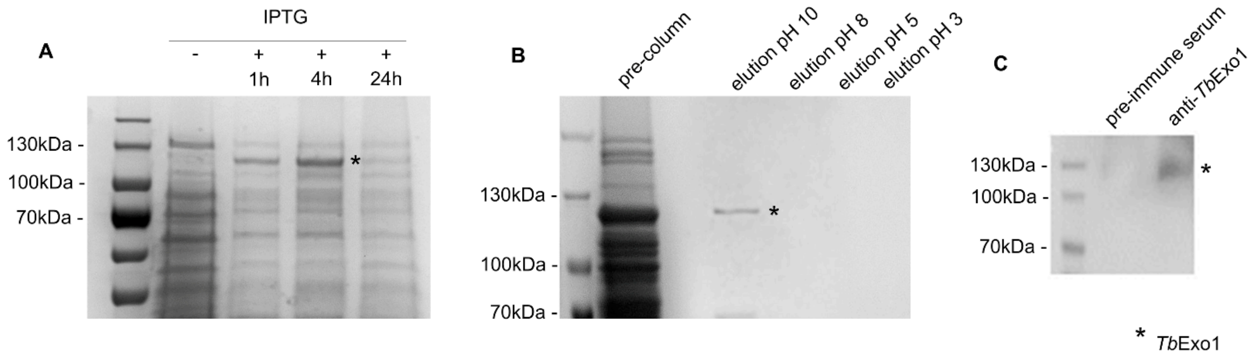

Supplementary Figure 3. Expression, purification and antisera generated against r*Tb*Exo1. A) rTbExo1 (~116 kDa) was expressed in the presence of IPTG in a prokaryotic system. B) rTbExo1 was purified using a nickel column and eluted with a linear gradient of pH. C) Protein extracts from *T. brucei* cells were submitted to SDS-PAGE and transferred onto nitrocellulose membranes that were incubated with anti-rTbExo1. (*) represents the rTbExo1.

Uncropped westerns

From Figure 4:

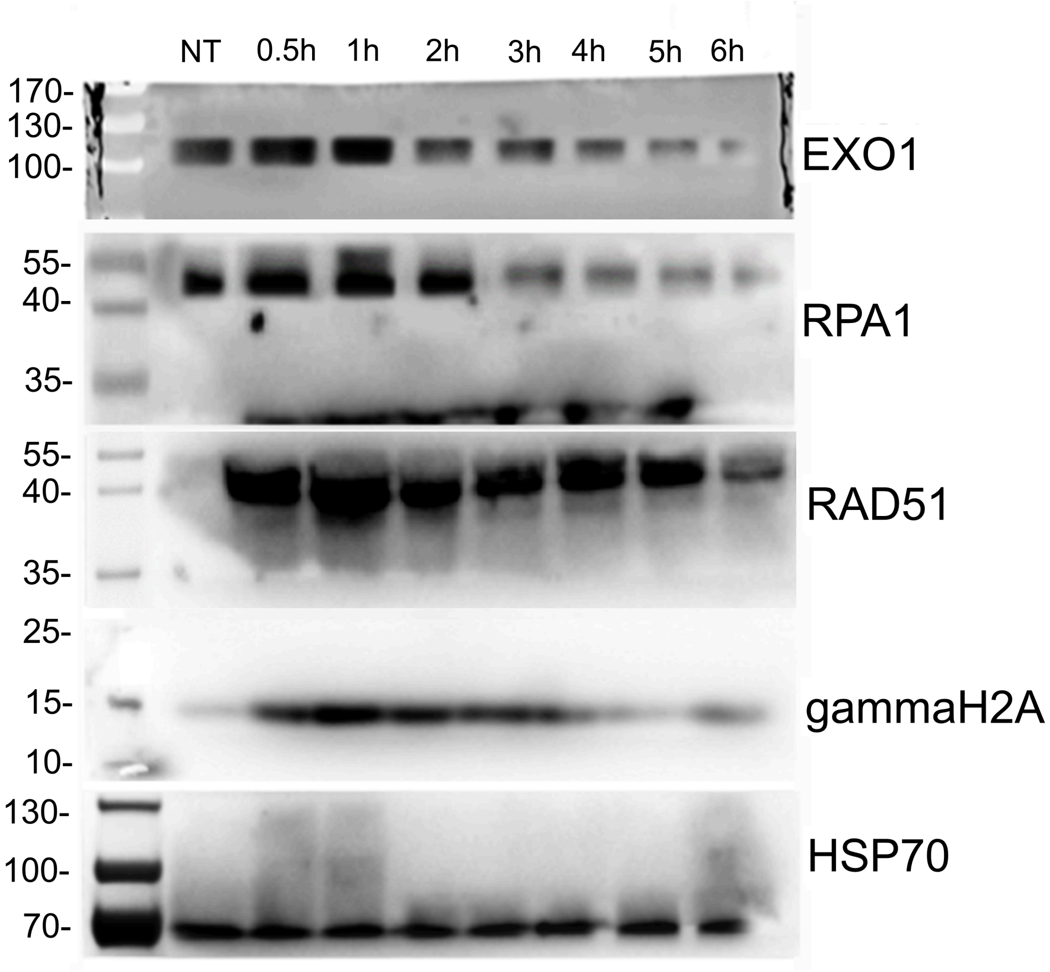

Figure 4. WB analysis of the total protein extract after 50 Gy of IR exposure. HSP70 protein was used as an internal control.

From Figure 5:

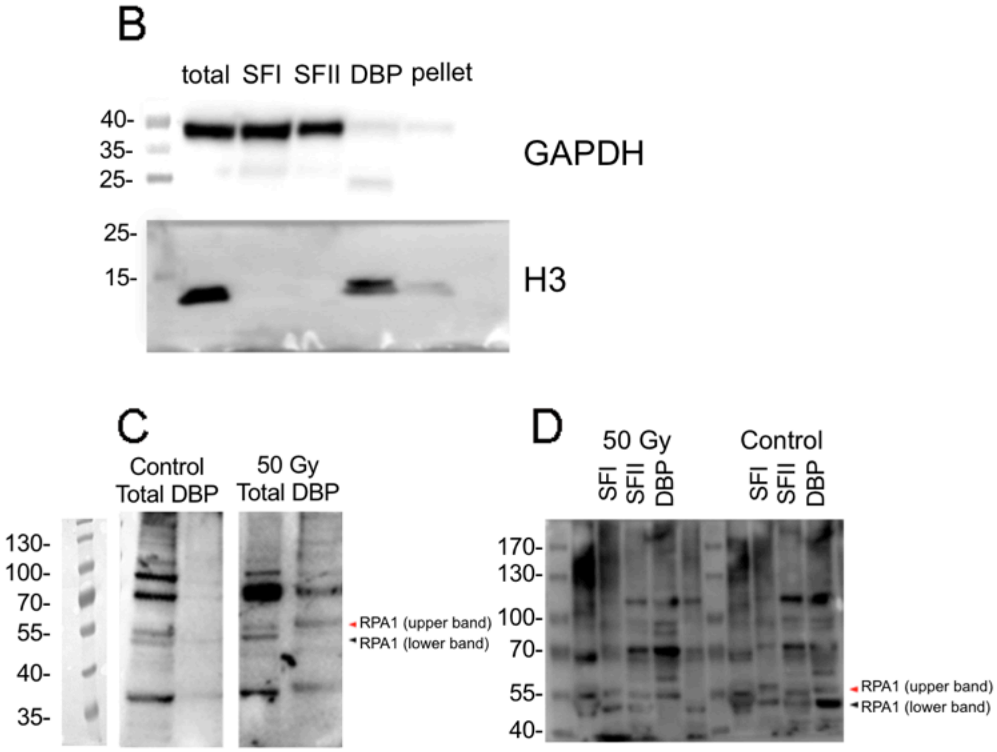

Figure 5. B) The GAPDH protein was used as an internal control for the soluble protein fraction (SFI and II). The H3 protein was used as an internal control for the DNA-bound protein fraction (DBP). **C and D)** Molecular weight migration profile of RPA1 in total, soluble protein fraction (SFI and II) and DNA bound protein (DBP) extracts after IR treatment.

From Figure 6:

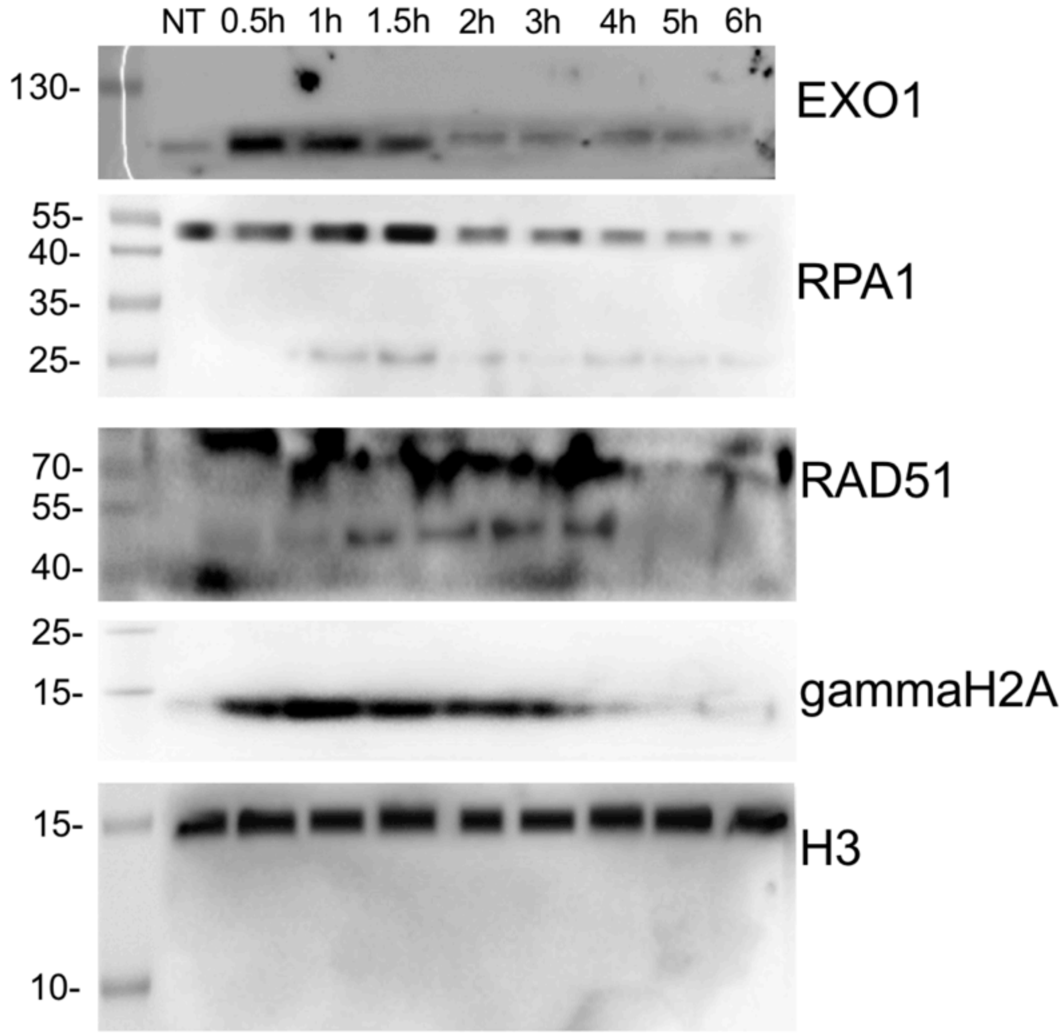

Figure 6. WB analysis of DNA-bound proteins (DBP) derived from the fractionation assay after IR treatment. The H3 protein was used as an internal control.

From Figure S1:

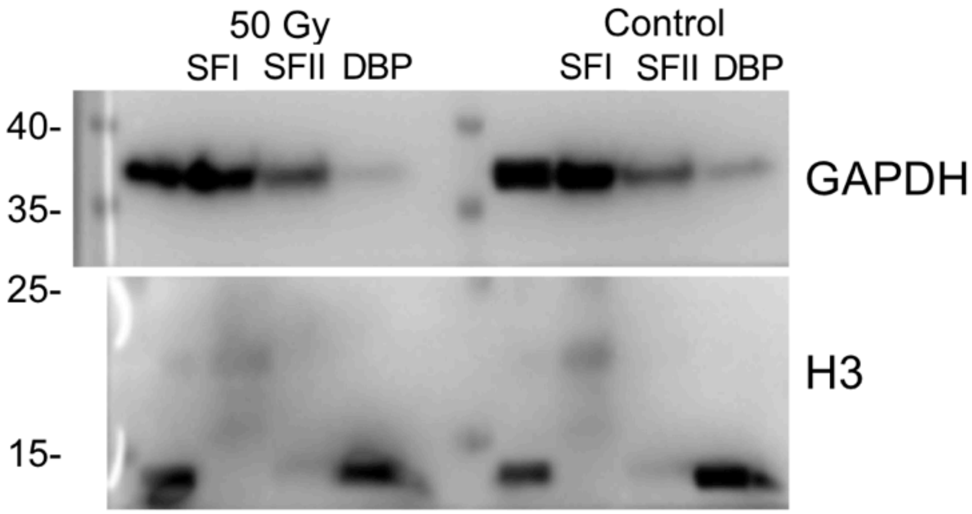

Supplementary Figure 1. Cells were exposed to IR (50 Gy) and treated with lyses buffer in order to obtain soluble proteins (soluble fraction – SF). After a second round of treatment with lyses buffer, pellets were digested with DNAse in order to get DNA bound proteins (DBP). Samples were then submitted to SDS-PAGE, transferred to nitrocellulose membrane and incubated with anti-GAPDH and with anti-histone H3.

From Figure S2:

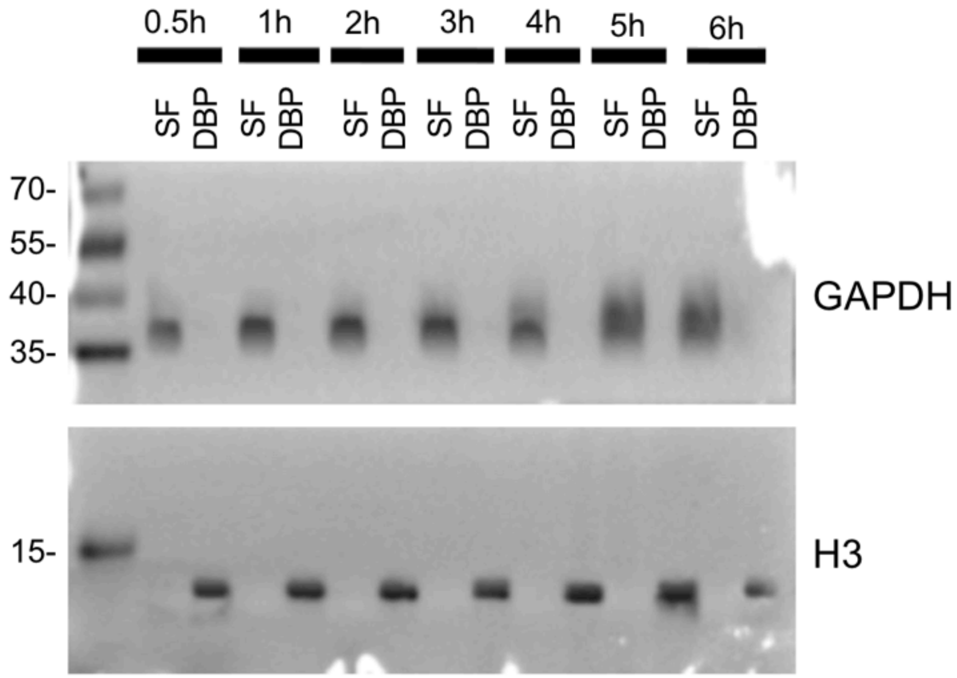

Supplementary Figure 2. Cells were exposed to IR and different times of exposition cells were treated with lyses buffer in order to obtain soluble proteins (soluble fraction – SF). After a second round of treatment with lyses buffer, pellets were digested with DNAse in order to get DNA bound proteins (DBP). Samples were then submitted to SDS-PAGE, transferred to nitrocellulose membrane and incubated with anti-GAPDH and with anti-histone H3.

From Figure S3:

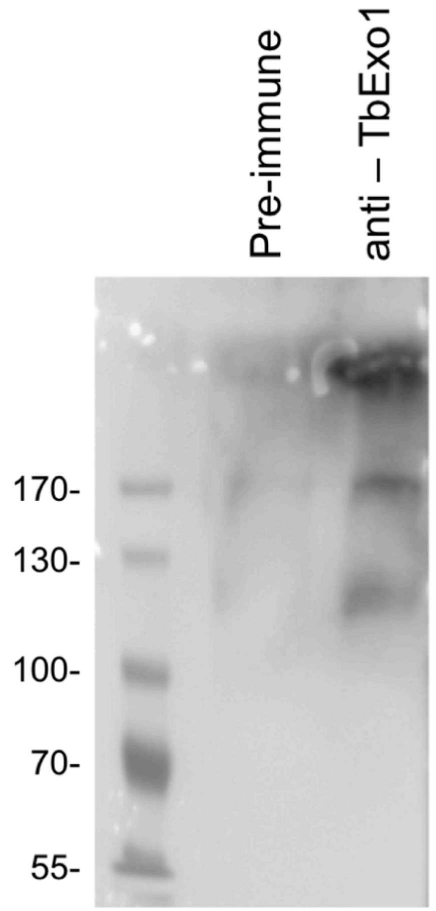

Supplementary Figure 3. Protein extracts from *T. brucei* cells were submitted to SDS-PAGE and transferred onto nitrocellulose membranes that were incubated with anti-rTbExo1.
